# Self‐Reported Plastic‐Related Occupational Scenarios and Perceived Support Priorities Among Operating‐Room Nursing Staff: A Multicentre Cross‐Sectional Survey

**DOI:** 10.1155/jonm/7475740

**Published:** 2026-10-17

**Authors:** Jie Yang, Panyu Chen, Zhihui Huang, Lin Yang, Qian Feng, Xiaoli Zhong, Qinghua Zhang, Junhong Zhou, Jun Cheng, Meng Shi, Qihao Chen, Yongqiong Tan, Yonghua Chen

**Affiliations:** ^1^ Division of Pancreatic Surgery Department of General Surgery West China Hospital Sichuan University Chengdu Sichuan 610041 China scu.edu.cn; ^2^ Department of Hepatobiliary and Pancreatic Surgery Gaoxin Hospital Affiliated to West China Second School of Medicine Sichuan University Chengdu Sichuan 610095 China scu.edu.cn; ^3^ Operating Room West China Hospital of Sichuan University/West China School of Nursing Sichuan University Chengdu Sichuan 610041 China scu.edu.cn; ^4^ Operating Room West China Tianfu Hospital Sichuan University Chengdu Sichuan 610213 China scu.edu.cn; ^5^ West China School of Medicine West China Hospital of Sichuan University Chengdu Sichuan 610041 China wchscu.cn; ^6^ Department of Hepatobiliary Surgery Deyang People’s Hospital Deyang Sichuan 618000 China; ^7^ Nursing Department Deyang People’s Hospital Deyang Sichuan 618000 China; ^8^ Anesthesia & Operation Department Sichuan University Affiliated Chengdu Second People’s Hospital Chengdu Sichuan 610017 China; ^9^ Department of Operating Room Nursing West China Second University Hospital Sichuan University Chengdu Sichuan 610041 China scu.edu.cn; ^10^ Key Laboratory of Birth Defects and Related Diseases of Women and Children (Sichuan University) Ministry of Education Chengdu Sichuan 610041 China meb.gov.tr; ^11^ Department of General Surgery West China Tianfu Hospital of Sichuan University Chengdu Sichuan 610213 China

**Keywords:** microplastics, nursing management, operating rooms, shift work, sustainable surgery

## Abstract

**Aim(s):**

To examine work‐context associations with self‐reported plastic‐related occupational scenario burden and perceived support priorities among operating‐room nursing staff.

**Background:**

Operating rooms rely heavily on plastic‐containing disposable materials and smoke‐generating workflows, but nursing‐management evidence on how staff report these occupational scenarios remains limited.

**Method(s):**

This exploratory multicentre cross‐sectional survey included 448 eligible nursing personnel from nine recruitment groups in China (eight hospital‐based; one visiting‐nurse training cohort); 415 frontline staff formed the analytic population. The principal outcome was a 0–100 formative occupational scenario‐burden index. Adjusted linear regression included recruitment‐group fixed effects, with alternative index definitions and binary distribution‐based outcomes examined as sensitivity analyses.

**Results:**

Among 415 frontline operating‐room nursing staff, the mean occupational scenario‐burden score was 50.7 (SD 12.4); 408 participants had complete data for the principal regression. Night‐shift status was associated with a 3.86‐point higher score (HC3 95% CI 0.96–6.76), and this association remained positive across the principal sensitivity analyses. Orthopaedic involvement was associated with a 4.93‐point higher score in the original index, although the estimate attenuated after specialty‐specific content was removed. The most frequently endorsed support priorities were rest‐area water infrastructure (69.9%), meal‐container reform (63.6%) and ventilation/smoke‐management optimisation (63.4%).

**Conclusion(s):**

Night‐shift work was associated with higher self‐reported occupational scenario burden, with positive point estimates across the principal sensitivity analyses, whereas the specialty‐related association was more sensitive to index composition. These findings identify organisational priorities for review and provide a basis for prospective studies integrating validated scenario measures with objective exposure assessment.

**Implications for Nursing Management:**

Staff‐endorsed priorities provide a practical agenda for nurse managers to review workflows, smoke‐control practices, procurement, rest‐area infrastructure and night‐shift support and to select priorities for prospective evaluation.

## 1. Introduction

Microplastics and other plastic‐associated environmental contaminants have raised increasing public‐health concern. Human exposure may occur through ingestion, inhalation and contact with plastic‐containing products, although the relative contribution of individual sources and the health significance of many exposure pathways remain uncertain [1, 2]. Healthcare depends heavily on disposable and plastic‐containing materials, and operating rooms are among the most resource‐intensive clinical environments [3–5]. Surgical smoke is also a recognised occupational concern because energy devices generate heterogeneous particulate matter and chemical by‐products [6, 7].

Operating‐room nurses are central to these processes because they prepare sterile materials, open packages, handle disposable supplies, maintain sterile fields, assist smoke‐related workflows and support room turnover. Existing perioperative sustainability research has focused mainly on waste generation, environmental impact and clinicians’ attitudes towards greener practice [3–5, 8], whereas less attention has been paid to how nursing staff report plastic‐related work scenarios, how those reports vary with work context and which organisational responses they prioritise. Broader nursing research suggests that workload, scheduling, staffing, leadership and organisational resources shape nurses’ work experiences and perceived care quality [9, 10]. Individual perceptions and behavioural intentions may also influence how staff recognise and respond to environmental concerns, for which the theory of planned behaviour provides a useful conceptual framework [11, 12].

Against this background, we conducted an exploratory multicentre cross‐sectional survey of operating‐room nursing personnel in China. We aimed to characterise self‐reported plastic‐related occupational scenarios, examine their associations with work‐context factors using a formative occupational scenario‐burden index and identify staff‐perceived institutional support priorities. We also explored cognitive‐behavioural measures and tested the robustness of the occupational findings across alternative index and model specifications.

## 2. Methods

This exploratory multicentre cross‐sectional survey examined self‐reported plastic‐related occupational scenarios, cognitive‐behavioural measures and perceived institutional support priorities among operating‐room nursing personnel recruited in China. The conceptual framework organised work‐context, daily‐life and cognitive‐behavioural variables (Figure 1). Reporting followed the Strengthening the Reporting of Observational Studies in Epidemiology (STROBE) guidance for cross‐sectional studies [13] and applicable Checklist for Reporting Results of Internet E‐Surveys (CHERRIES) items for the online component [14]. The study was designed for exploratory estimation; inference, therefore, emphasised effect sizes, confidence intervals and sensitivity to plausible index and model specifications.

**FIGURE 1:**
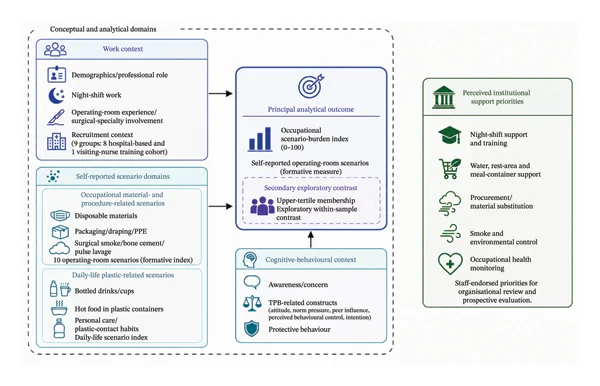
Conceptual and analytical framework of the study. Work‐context and self‐reported scenario domains were examined in relation to the principal continuous occupational scenario‐burden index; upper‐tertile membership provided a secondary sensitivity contrast. Cognitive‐behavioural measures and perceived institutional support priorities were parallel analytical domains. Arrows represent analytical associations within the cross‐sectional framework. PPE, personal protective equipment; TPB, theory of planned behaviour.

### 2.1. Setting, Participants and Recruitment

Data were collected from 2 December 2025 to 23 March 2026 using convenience sampling. Recruitment context was represented by nine groups: eight hospital‐based groups from tertiary Grade A hospitals in China, most located in southwestern China, and one visiting‐nurse training cohort comprising nurses from multiple home institutions. The visiting‐nurse cohort represented a single recruitment context; home institution was not used to define separate recruitment groups. Recruitment group was used as the contextual unit for adjustment in the analyses. Eligible participants were nursing personnel working in or affiliated with operating‐room services who provided written or electronic informed consent; clearly nonnursing professional categories were excluded. Professional nursing registration status was not separately recorded. The frontline analytic population comprised personnel reporting direct involvement in routine operating‐room nursing, including circulating, scrub/instrument or anaesthesia‐monitoring roles. Nursing personnel with managerial, quality, coordination, scheduling or related responsibilities were classified as frontline when they also reported direct operating‐room duties. No minimum weekly operating‐room frequency was specified. Local investigators or operating‐room nursing managers distributed study notices through departmental meetings or communication groups. Managers were not present during questionnaire completion, did not collect individual responses and could not identify individual participants from study responses. Participation or nonparticipation did not affect scheduling, performance appraisal or employment evaluation; participation was voluntary, and no incentive was provided. Participation is reported using the 449 formally submitted questionnaires because study records did not preserve a unique eligible/invited denominator. The online platform recorded 782 access events, including repeat scans or reopened questionnaires; these were retained as platform‐use information rather than unique‐participant counts. A recruitment‐yield approach was used, with all eligible submissions during the predefined collection period included in the analysis.

### 2.2. Questionnaire Administration, Duplicate Control and Development

Most recruitment groups used a structured questionnaire administered through Jinshuju, a Chinese online survey platform, via quick‐response code or web link. Two hospital‐based recruitment groups (Groups D and F) used paper self‐administered questionnaires exclusively. The paper form contained the same items and response options as the electronic version, was completed without interviewer administration and was entered into the electronic database with independent second‐person verification. The Chinese questionnaire used for data collection is provided as Supporting File 1 (Chinese questionnaire, anonymised). Supporting File 2 provides an English reporting translation; this English version was prepared for reporting purposes only and was not used for data collection. Formally submitted questionnaires underwent eligibility and duplicate checks. Designed nonscorable responses were excluded from the relevant item denominator, model‐specific missing covariates were handled by complete‐case analysis, and partial item completion alone did not trigger questionnaire exclusion. The archived platform export preserves response data but not the original item‐by‐item required/optional settings; completeness was therefore evaluated from observed data and the documented scoring rules.

During data collection, the online platform was configured to prevent multiple independent submissions by the same participant; repeated access allowed respondents to review or update the existing record rather than generate a second questionnaire. After data collection closed, new submissions and further editing were disabled; respondents could only view their own submitted records. Available IP/mobile‐phone‐linked platform information, staff/badge numbers, abbreviated identifiers and manual verification were used for duplicate checking. Access to identifying information was limited to designated study investigators responsible for data‐quality verification. No duplicate questionnaire records were identified among the 449 formally submitted records entering data cleaning. Identifying information used for checking was removed or de‐identified before analysis; the manuscript‐facing dataset used anonymised recruitment‐group codes. Instrument development combined operating‐room workflow mapping, literature‐informed questionnaire design, qualitative expert review and a 15‐participant usability pilot [15]. Three experts (two nursing experts and one with surgical/clinical and statistical expertise) reviewed item relevance, wording, clarity, response options and applicability to operating‐room practice. The pilot assessed clarity, completion time, usability and consistency of interpretation; pilot participants were excluded from the formal survey sample. This initial development stage provided preliminary evidence regarding item relevance and usability, while formal content‐validity indexing, cognitive interviewing, construct validation and external validation remain priorities for subsequent measurement work.

### 2.3. Scenario‐Burden Indices

The occupational scenario‐burden index comprised 10 operating‐room items (C4.1–C4.10) spanning general material handling and packaging, apparel/draping, smoke or particulate‐related situations, and specialty‐specific tasks including bone cement/pulse lavage. The items intentionally sampled heterogeneous work situations within a formative work‐scenario framework. Supporting Table S1 maps each item to its conceptual domain, potential material or pathway represented, principal construct captured, applicability, scoring and key limitations. Ordinal responses were recoded so that higher values represented more frequent or intensive reporting. The original occupational index was calculated as 100 multiplied by the sum of valid item scores divided by the corresponding maximum possible sum for those valid items. Missing or nonscorable responses were excluded from both numerator and denominator. Among 415 frontline participants, 345 contributed 10 valid items, 61 contributed nine, and nine contributed eight; thus, all calculated occupational scores contained at least eight valid items. A daily‐life scenario‐burden index was constructed analogously from 10 items concerning drinking containers, bottled drinks, disposable cups, hot food in plastic containers or bags and selected personal‐care/plastic‐contact practices. The indices were specified as formative summaries because their component scenarios need not covary or reflect a single latent construct [16]; Cronbach’s alpha was, therefore, reserved for reflective multi‐item measures. Higher index values represent more frequent or intensive reporting across the included scenarios. Environmental exposure, internal biological burden and health outcomes were treated as separate measurement domains requiring objective assessment. Sensitivity analyses examined person‐specific denominators, heterogeneous item content and equal‐weighting/compensability assumptions.

### 2.4. Cognitive‐Behavioural Measures, Support Priorities and Open‐Ended Comments

Awareness and concern were analysed as continuous numeric ratings. Theory‐of‐planned‐behaviour‐informed measures [11, 12] included attitude, norm pressure, peer environmental influence, perceived behavioural control, behavioural intention and protective behaviour. Internal consistency of reflective multi‐item constructs was assessed using Cronbach’s alpha, standardised alpha and average inter‐item correlation [17]. At the analysis stage, the attitude score was refined after the original four‐item version showed poor internal consistency: the reverse‐worded health‐dismissal item was removed, leaving three aligned items. Peer environmental influence was analysed separately from the three‐item norm‐pressure score. These post hoc scale refinements are documented transparently in Supporting Table S2. Perceived institutional support priorities were assessed using a multiple‐response item covering rest‐area water infrastructure, canteen/meal‐container reform, ventilation and smoke‐management optimisation, occupational‐health monitoring, nonplastic medical alternatives, night‐shift/duty support and training. Endorsement frequencies were used to rank staff‐perceived organisational priorities for further assessment and prospective evaluation. Substantive open‐ended comments were coded by two researchers using descriptive content analysis [18, 19], with disagreements resolved through discussion and multiple themes allowed per comment. The 26 substantive comments were summarised as a descriptive supplement to the structured survey.

### 2.5. Analytical Outcomes and Model Hierarchy

The analytic hierarchy was finalised during revision, with the continuous 0–100 occupational scenario‐burden index designated as the principal analytical outcome because it retains the full information in the score. Upper‐tertile membership was retained as an exploratory sensitivity analysis: scores > 54.0541 were classified in the upper group, with ties at 54.0541 retained in the lower group. A second binary sensitivity used the frontline‐specific 75th percentile (> 58.8235), again retaining ties at the cut‐off in the lower group. Both cut‐offs were sample‐derived and were used as within‐sample sensitivity definitions of the continuous principal outcome; additional robustness analyses were likewise treated as exploratory. Night‐shift status was analysed primarily as a binary work‐context variable. An exploratory analysis additionally classified night‐shift frequency as no night shift, < 5 night shifts/month or ≥ 5 night shifts/month using the structured shift‐pattern and monthly‐frequency items. Where monthly frequency was unavailable, shift‐pattern information was used to classify clearly day‐only schedules; unresolved classifications were treated as missing. Sensitivity analyses applied a more conservative classification rule and, separately, were restricted to participants with directly recorded night‐shift frequency.

### 2.6. Statistical Analysis

Continuous variables were summarised as means (standard deviations) and categorical variables as *n* (%). Two‐sample *t* tests and chi‐square tests characterised observed group patterns where appropriate; index robustness was evaluated separately through the sensitivity‐analysis framework. The principal analysis used ordinary least‐squares regression with the continuous occupational score as the outcome. Model 1 was a parsimonious work‐context model including age, sex, operating‐room work years, night‐shift status, orthopaedic‐surgery involvement, interventional‐surgery involvement and recruitment group as a fixed effect. Recruitment‐group fixed effects adjusted for recruitment context, with their coefficients retained as adjustment parameters. Model 2 additionally included the daily‐life score, concern, attitude, norm pressure, peer environmental influence, perceived behavioural control, behavioural intention and protective behaviour; these continuous variables were standardised before modelling. Model 2 was specified as an exploratory extension to examine whether adding daily‐life and cognitive‐behavioural domains altered the Model 1 associations. Because all domains were measured concurrently, their coefficients were interpreted as exploratory explanatory associations. Model diagnostics included residual‐versus‐fitted and related residual plots, Breusch–Pagan heteroskedasticity tests, Cook’s D and leverage influence screening, case‐deletion sensitivity, functional‐form checks and multicollinearity assessment. Conventional estimates were accompanied by HC3 heteroskedasticity‐consistent confidence intervals and *p* values, where relevant. For concern and attitude, progressive same‐sample models were supplemented by correlation, generalised variance inflation factor (GVIF) and nonlinear functional‐form checks. Sensitivity analyses examined alternative index definitions and subdomains, including removal of specialty‐specific content, equal‐item weighting, removal of the two nonstandard/context‐sensitive scoring items, complete common‐item samples and same‐sample comparisons where possible. Upper‐tertile and upper‐quartile outcomes were modelled with logistic regression using the same Model 1 and Model 2 structures. Because the binary outcome was common, model‐standardised probabilities and absolute contrasts supplemented odds ratios. Binary‐model stability was assessed using parameter counts, sparse‐data checks, discrimination, calibration, bootstrap resampling and ridge‐regression sensitivity. Recruitment‐context sensitivity was examined using recruitment‐group random‐intercept and leave‐one‐group‐out analyses. Survey‐mode sensitivity excluded the two paper‐only groups (D and F). Because administration mode was nested within recruitment group, this analysis specifically assessed robustness within the online component; separate mode effects were not identifiable. Principal models used complete‐case analysis for required covariates [20]. No flat‐response exclusions were applied. The exploratory inference framework prioritised effect estimates, confidence intervals and sensitivity to plausible model and index specifications. Analyses were conducted in R version 4.6.0 using two‐sided tests.

### 2.7. Ethical Considerations

The ethics committee of the lead institution approved the study on 4 November 2025 (No. 2025‐066). Participants received information about the study purpose, voluntary participation, confidentiality and intended data use before providing written or electronic informed consent. Information used for duplicate checking was retained only for data‐quality verification and removed before analysis; the analytic dataset was de‐identified before analysis and reporting.

## 3. Results

Of 449 formally submitted questionnaires, one response from a nonnursing doctor was excluded, leaving 448 eligible nursing personnel. Thirty‐three respondents without direct frontline operating‐room nursing duties were not included in the frontline analyses, leaving 415 frontline operating‐room nursing staff; 408 had complete data for the principal regression models after seven participants with unresolved night‐shift status were excluded. For the secondary distribution‐based analysis, 138 participants were above the occupational‐score upper‐tertile cut‐off and 277 were in the lower two tertiles (Figure 2). Participants were recruited through nine recruitment groups: eight hospital‐based groups and one visiting‐nurse training cohort comprising nurses from multiple home institutions. Recruitment‐group contributions to the frontline sample ranged from 15 to 118 participants. Groups D and F used paper self‐administered questionnaires exclusively, contributing 44 and 29 eligible respondents (40 and 29 frontline participants), respectively; the remaining groups used online administration. Recruitment‐group contributions and role/specialty composition are detailed in Supporting Tables S3 and S4. Among frontline participants, mean age was 31.4 years (SD 6.3), 349 (84.1%) were female, 311 (74.9%) reported night‐shift work, and 161 (38.8%) reported orthopaedic‐surgery involvement (Table 1).

**FIGURE 2:**
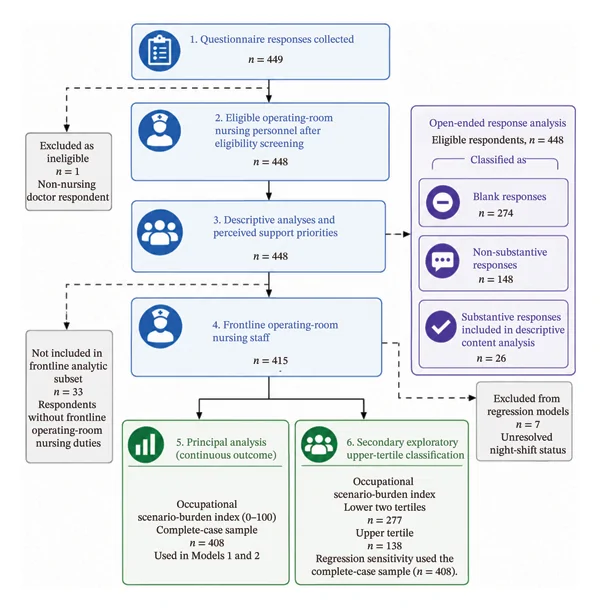
Flow of participants and analytical samples. Of 449 submitted responses, one nonnursing doctor was excluded, leaving 448 eligible nursing personnel. The frontline subset comprised 415 staff; 408 had complete data for the principal regression models after seven with unresolved night‐shift information were excluded. For the exploratory upper‐tertile classification among all 415 frontline staff, 277 were in the lower two tertiles and 138 in the upper tertile; among the 408 regression complete cases, 135 were upper‐tertile events. Open‐ended responses were blank (*n* = 274), nonsubstantive (*n* = 148) or substantive (*n* = 26).

**TABLE 1:** Participant characteristics of frontline operating‐room nursing staff.

Characteristic	Overall, *n* = 415
Age, years	31.4 (6.3)
Body mass index (kg/m^2^)	22.3 (5.3)
Female sex	349 (84.1)
Male sex	66 (15.9)
Operating‐room work years: < 1 year	20 (4.8)
Operating‐room work years: 1–3 years	100 (24.1)
Operating‐room work years: 4–6 years	88 (21.2)
Operating‐room work years: 7–10 years	86 (20.7)
Operating‐room work years: > 10 years	121 (29.2)
Professional title: nurse	58 (14.0)
Professional title: senior nurse	221 (53.3)
Professional title: supervisor nurse	123 (29.6)
Professional title: associate chief nurse or above	13 (3.1)
Shift pattern: day‐only/nonregular daytime	77 (18.6)
Shift pattern: rotating/extended shift	334 (80.5)
Shift pattern: unclear/other	4 (1.0)
Any night shift: no	97 (23.4)
Any night shift: yes	311 (74.9)
Any night shift: missing	7 (1.7)
Circulating nurse role	286 (68.9)
Scrub/instrument nurse role	284 (68.4)
Anaesthesia‐monitoring nurse role	10 (2.4)
Operating‐room management/quality role	27 (6.5)
General‐surgery involvement	233 (56.1)
Orthopaedic‐surgery involvement	161 (38.8)
Interventional‐surgery involvement	46 (11.1)
Neurosurgery involvement	106 (25.5)
Gynaecology/urology involvement	212 (51.1)

*Note:* Data are mean (SD) or *n* (%). Professional roles and surgical specialties were multiple‐response items.

### 3.1. Scenario‐Burden Indices and Questionnaire Measures

The mean occupational scenario‐burden score was 50.7 (SD 12.4), and the mean daily‐life scenario‐burden score was 38.3 (SD 13.8). The distributions of both indices, including the exploratory upper‐tertile and upper‐quartile cut‐offs for the occupational score, are shown in Supporting Figure S1. For the 10‐item occupational score, 345 participants (83.1%) contributed all 10 valid items, 61 (14.7%) contributed nine, and nine (2.2%) contributed eight; all 415 frontline participants, therefore, had a calculable occupational score. Nonscorable responses occurred most often for sterile‐package opening (C4.4; *n* = 50) and laparoscopic smoke management (C4.9; *n* = 29), including nine participants with both items nonscorable (Supporting Table S5). Component‐item distributions for the exploratory upper‐tertile comparison are shown in Supporting Table S6.

### 3.2. Principal Continuous Regression Analysis

In the principal continuous Model 1 (*n* = 408), night‐shift status was associated with a 3.86‐point higher occupational scenario‐burden score (HC3 95% CI 0.96–6.76) and orthopaedic‐surgery involvement with a 4.93‐point higher score (HC3 95% CI 2.42–7.44) (Table 2). Interventional‐surgery involvement, age, sex and operating‐room work years had confidence intervals that included zero. Recruitment‐group coefficients are reported separately in Supporting Table S7. In the extended exploratory Model 2, the estimates for night‐shift status (*β* = 3.17; HC3 95% CI 0.22–6.12) and orthopaedic involvement (*β* = 4.90; 2.40–7.41) remained positive. The daily‐life scenario‐burden index and the other cognitive‐behavioural variables had HC3 confidence intervals that included zero. Concern and attitude yielded positive conventional ordinary‐least‐squares estimates in Model 2, but their HC3 intervals also included zero, indicating sensitivity of these secondary findings to the variance specification (Supporting Table S7).

**TABLE 2:** Principal continuous regression models for self‐reported occupational scenario burden.

Associated factor	Model 1 β (HC3 95% CI)	*p*	Model 2 β (HC3 95% CI)	*p*
Age, per year	0.00 (−0.31 to 0.30)	0.982	0.00 (−0.30 to 0.30)	0.999
Male sex	2.77 (−0.39 to 5.93)	0.085	2.24 (−0.96 to 5.43)	0.170
Operating‐room work years, per ordinal category	−0.40 (−1.81 to 1.02)	0.581	−0.22 (−1.66 to 1.23)	0.767
Any night shift	3.86 (0.96 to 6.76)	0.009	3.17 (0.22 to 6.12)	0.035
Participation in orthopaedic surgery	4.93 (2.42 to 7.44)	< 0.001	4.90 (2.40 to 7.41)	< 0.001
Participation in interventional surgery	1.94 (−1.61 to 5.49)	0.284	1.75 (−1.77 to 5.27)	0.330
Daily‐life scenario‐burden index, per 1 SD	—	—	0.71 (−0.50 to 1.92)	0.251
Concern score, per 1 SD	—	—	1.41 (−0.07 to 2.88)	0.062
Attitude score, per 1 SD	—	—	2.08 (−0.22 to 4.38)	0.077
Norm pressure score, per 1 SD	—	—	0.82 (−0.75 to 2.40)	0.306
Peer environmental influence, per 1 SD	—	—	−1.09 (−2.81 to 0.63)	0.213
Perceived behavioural control, per 1 SD	—	—	−0.86 (−3.02 to 1.30)	0.433
Behavioural intention, per 1 SD	—	—	−1.91 (−4.32 to 0.50)	0.119
Protective behaviour, per 1 SD	—	—	0.11 (−1.26 to 1.47)	0.879

*Note:* Outcome: original 10‐item occupational scenario‐burden index (0–100); *n* = 408 in both models. Model 1 included age, sex, operating‐room work years, night‐shift status, orthopaedic involvement, interventional involvement and recruitment‐group fixed effects. Model 2 additionally included the daily‐life index and cognitive‐behavioural variables. Point estimates are ordinary least‐squares coefficients; 95% CIs and *p* values use HC3 heteroskedasticity‐consistent standard errors. Recruitment‐group coefficients are reported in Supporting Table S7.

### 3.3. Index‐Construction Sensitivity and Orthopaedic Circularity

Alternative scoring analyses distinguished sensitivity to numerical weighting from sensitivity to item content (Supporting Tables S8 and S9). Equal‐item weighting and removal of C4.4/C4.9 produced estimates close to the original score, supporting robustness of the principal night‐shift result and the original orthopaedic estimate to these numerical and coding choices. In contrast, removing specialty‐specific content materially attenuated the orthopaedic estimate. The Model 1 coefficient decreased from 4.93 points (HC3 95% CI 2.42–7.44) with the original index to 2.47 (−0.14 to 5.09) after removing C4.10 and to 1.83 (−1.65 to 5.32) with the six‐item general/common index; both alternative estimates had HC3 intervals that included zero. Same‐sample comparisons showed the same pattern, indicating that the attenuation was not explained solely by loss of participants. Night‐shift coefficients remained positive across the principal alternative score definitions, although magnitude and precision varied. In the six‐item general/common analysis, for example, the estimate was 4.14 points (HC3 95% CI −0.01 to 8.28); the corresponding same‐sample original‐score estimate was 3.00 (−0.16 to 6.15).

### 3.4. Concern, Attitude and Night‐Shift Frequency Analyses

Progressive same‐sample models clarified the concern pattern (Supporting Table S10). The coefficient was negative before recruitment‐group adjustment and became positive after recruitment group was introduced, reaching +1.41 points per SD in the full model; its HC3 95% CI crossed zero (−0.07 to 2.88). Attitude remained positive across stages and was also imprecise under HC3 inference. Correlation and GVIF diagnostics indicated low multicollinearity, and functional‐form checks showed no clear nonlinearity. In the exploratory night‐shift‐frequency analysis, adjusted occupational scenario‐burden scores were higher for both < 5 and ≥ 5 night shifts/month than for no night shift; the ≥ 5/month contrast was 5.45 points (HC3 95% CI 1.65–9.26; Supporting Table S11). The sensitivity analysis restricted to directly recorded frequency responses had a small no‐night reference group and substantially wider intervals. The frequency analysis, therefore, provided directional consistency with the binary night‐shift finding, while its precision was insufficient to characterise a dose‐response gradient.

### 3.5. Exploratory Binary Models and Robustness Analyses

The upper‐tertile analysis provided a secondary exploratory sensitivity (Supporting Table S12). In Model 1, night‐shift status was associated with upper‐tertile membership (OR 2.16, 95% CI 1.16–3.99), corresponding to model‐standardised probabilities of 22.0% without night‐shift work and 36.3% with night‐shift work.

Using the frontline‐specific upper‐quartile threshold of 58.8235, Model 1 results were directionally similar (night‐shift OR 3.84, 95% CI 1.67–8.83; orthopaedic OR 2.47, 1.42–4.31). Binary‐model diagnostics showed 14 and 22 nonintercept parameters in Models 1 and 2 (events per parameter 9.64 and 6.14, respectively); bootstrap‐corrected area under the receiver operating characteristic curves (AUCs) were 0.647 and 0.641 and corrected calibration slopes were 0.750 and 0.643 (Supporting Table S13). Ridge sensitivity shrank the night‐shift and orthopaedic coefficients. These diagnostics supported exploratory use of the binary models and continued to indicate limited predictive stability. The nested upper‐tertile Model 1 versus Model 2 fit comparison showed no improvement after adding daily‐life and cognitive‐behavioural variables (Akaike information criterion 499.81 vs. 510.44; likelihood‐ratio deviance difference 5.36, df = 8, *p* = 0.718; Supporting Table S13). Recruitment‐group random‐intercept and leave‐one‐group‐out analyses yielded positive point estimates for night shift and orthopaedic involvement across specifications, although some intervals crossed the null (Supporting Table S14), supporting directional consistency rather than uniform statistical precision across recruitment contexts. In the online‐only complete‐case sample, the night‐shift coefficient remained positive (*β* = 3.67; HC3 95% CI 0.34–7.01). The orthopaedic estimate again attenuated after removal of C4.10 (*β* = 2.04; −0.78 to 4.85; Supporting Table S15).

### 3.6. Perceived Institutional Support Priorities and Open‐Ended Comments

Among all 448 eligible respondents, the most frequently endorsed support options were water infrastructure in operating‐room rest areas (69.9%), canteen/meal‐container reform (63.6%), ventilation and smoke‐management optimisation (63.4%), occupational‐health monitoring (62.5%), nonplastic medical alternatives (56.7%), night‐shift/duty support (51.8%) and microplastic‐related knowledge and skills training (50.2%); rankings were similar in the frontline subset (Table 3). Twenty‐six of 448 eligible respondents (5.8%) provided substantive open‐ended comments. Themes concerned education, material substitution, occupational‐health protection, policy/procurement, smoke control and food, water or rest‐area infrastructure. The comments were summarised descriptively in Supporting Table S16. Detailed regression diagnostics are summarised in Supporting Table S17. Heteroskedasticity diagnostics supported the use of HC3 inference, while influence and functional‐form sensitivity checks did not materially change the principal interpretation.

**TABLE 3:** Perceived institutional support priorities endorsed by respondents.

Staff‐endorsed support option	Eligible respondents, *n* = 448	Frontline nursing staff, *n* = 415
Water infrastructure in operating‐room rest areas	313 (69.9)	292 (70.4)
Canteen/meal‐container reform	285 (63.6)	263 (63.4)
Operating‐room ventilation and smoke‐management optimisation	284 (63.4)	262 (63.1)
Occupational‐health monitoring	280 (62.5)	263 (63.4)
Nonplastic medical alternatives	254 (56.7)	233 (56.1)
Night‐shift/duty support	232 (51.8)	218 (52.5)
Microplastic‐related knowledge and skills training	225 (50.2)	209 (50.4)
Other support options	9 (2.0)	7 (1.7)

*Note:* Data are *n* (%). Multiple selections were allowed; percentages, therefore, do not sum to 100%. ‘Occupational‐health monitoring’ was a broad questionnaire option and did not specify a predefined monitoring protocol.

## 4. Discussion

This exploratory multicentre survey identified a positive association between night‐shift status and higher self‐reported occupational scenario‐burden scores among frontline operating‐room nursing staff. Night‐shift point estimates remained positive across alternative score definitions and recruitment‐context sensitivity analyses, although precision varied. In contrast, the association with orthopaedic involvement was substantially attenuated when specialty‐specific index content was removed, with HC3 intervals including zero in the principal B9 and six‐item general/common analyses. This pattern indicates greater dependence on how the formative occupational score was constructed. Staff also endorsed a broad set of organisational support priorities spanning rest‐area infrastructure, meal‐container reform, ventilation and smoke management, occupational‐health monitoring, material alternatives, night‐shift support and training. Taken together, these findings characterise reported work‐context patterns and staff‐perceived organisational priorities; they should not be interpreted as evidence of objective microplastic exposure or occupational risk.

Night‐shift status is best interpreted as a broad work‐system marker rather than as a direct proxy for exposure. Night work may coincide with differences in workload, staffing, procedure mix, material throughput, working hours, opportunities for rest and access to organisational or environmental controls. Shift and night work are established nursing and occupational‐health concerns because they can affect fatigue, performance, recovery and organisational functioning [21–24]. In the present study, night‐shift point estimates remained positive across alternative index definitions, recruitment‐group sensitivity analyses and the online‐only analysis, although precision varied and some sensitivity intervals crossed zero. These results, therefore, support interpreting night‐shift status as an organisational context associated with reported scenario burden, rather than as evidence of a dose‐response relationship or a direct exposure mechanism. The exploratory frequency analysis was directionally compatible with the binary finding, although the directly recorded‐frequency sensitivity had a small no‐night reference group and wide confidence intervals.

The orthopaedic finding illustrates a different issue: the importance of content composition when using a formative occupational index. Equal‐item weighting and removal of the two nonstandard or context‐sensitive scoring items produced estimates close to those obtained with the original score, whereas removal of the bone‐cement/pulse‐lavage item and use of the six‐item general/common index substantially attenuated the orthopaedic coefficient. Same‐sample comparisons showed the same pattern, making loss of participants an unlikely explanation for the attenuation. Thus, specialty‐specific item content, rather than numerical weighting alone, contributed materially to the original association. Orthopaedic workflows may indeed involve material‐intensive tasks, protective equipment, smoke and aerosols [6, 7], but the present analysis cannot distinguish a genuine specialty‐context difference from overlap between the specialty variable and the scenarios included in the index. This sensitivity is, therefore, an important measurement consideration when interpreting specialty‐related coefficients derived from formative work‐scenario scores.

The daily‐life and cognitive‐behavioural findings require a different interpretation. The daily‐life scenario score provided little additional information in the adjusted models, while concern and attitude showed positive but imprecise associations under HC3 inference. Because concern, attitudes, perceived control, intention, behaviour and occupational scenario reporting were measured concurrently, their associations may reflect antecedent beliefs, consequences of work experience, awareness, reporting tendencies or shared measurement processes. Likewise, the weak conditional association with the daily‐life score does not establish discriminant validity between occupational and nonoccupational domains. The two indices differ in item content, opportunity structure, applicability and measurement error, and both were obtained from the same questionnaire. Accordingly, the present data do not support ranking work‐context and individual cognitive‐behavioural domains by causal or explanatory importance; formal construct validation will require independent measures and external criteria.

The support‐priority findings extend the regression results by identifying the organisational domains that respondents most often selected for attention. Water infrastructure in operating‐room rest areas, meal‐container reform, ventilation and smoke‐management optimisation, occupational‐health monitoring, nonplastic alternatives, night‐shift support, and training span workplace infrastructure, perioperative materials, environmental controls and staff support. These priorities are consistent with the practical constraints described in perioperative sustainability research, including infection‐control requirements, workflow demands, material availability and procurement structures [3–5, 8], and with broader nursing evidence linking workload, scheduling, organisational resources and leadership to nurses’ work experiences [9, 10]. They should be regarded as staff‐endorsed domains for organisational review and prospective evaluation rather than as interventions already shown to reduce exposure or improve health. The small set of substantive open‐ended comments broadly echoed several of these structured priorities, particularly education, material substitution, procurement, smoke control, occupational‐health protection and rest‐area infrastructure, but their self‐selected nature limits their interpretive weight.

## 5. Implications for Nursing Management

These findings identify several domains for multidimensional organisational review, including workflow, smoke‐control practices, procurement, rest and drinking‐water infrastructure, and night‐shift support. Nurse managers can use the reported scenario patterns to identify material‐intensive tasks and work contexts that merit closer assessment and can embed those assessments within existing occupational‐safety, infection‐prevention, environmental‐health and procurement processes. General control principles and established perioperative smoke‐safety guidance can inform prioritisation [25–27]. Material substitution, ventilation engineering, procurement policy and occupational‐health monitoring are inherently multidisciplinary and require nursing leadership alongside infection‐prevention teams, occupational‐health services, engineers, procurement and hospital management. Any alternative should preserve sterility and clinical performance [28]. In this survey, ‘occupational‐health monitoring’ functioned as a broad staff‐priority category; future implementation would require a defined protocol and validated measurement strategy. Smoke‐evacuation availability and adherence likewise warrant direct workflow assessment. Night‐shift and specialty contexts offer practical starting points for targeted organisational assessment. Prospective work‐system studies should measure staffing, workload, procedure mix, material throughput, shift characteristics and environmental‐control conditions alongside self‐reported experiences, with objective environmental sampling and, where validated and appropriate, biological assessment.

## 6. Limitations

Several limitations should be considered. First, the cross‐sectional design leaves temporal ordering unresolved, and residual confounding is possible because workload, case mix, staffing, working hours, specialty mix, material throughput and related organisational factors were not measured in sufficient detail. Concurrent self‐report of occupational scenarios, cognitive‐behavioural measures and support priorities also introduces potential recall, interpretation, social‐desirability and common‐method bias. Second, the occupational scenario‐burden index represents an initial formative measurement approach. Its components differ in content, frequency scale and applicability, and person‐specific denominators mean that some scores were based on different sets of valid items. The attenuation of the orthopaedic association after removing specialty‐specific content illustrates the sensitivity of some findings to index composition. Expert review and pilot testing provided preliminary evidence regarding item relevance and usability, but formal content, construct and external validation remain needed. The sample‐derived tertile and quartile contrasts and the parameter‐rich extended binary model were, therefore, retained only as secondary sensitivity analyses, with the continuous score remaining the principal outcome. Third, convenience sampling was concentrated in eight tertiary hospital‐based recruitment groups, mostly in southwestern China, together with one visiting‐nurse cohort comprising nurses from multiple home institutions, which limits generalisability. Because the visiting‐nurse cohort was a recruitment context rather than an institution‐level cluster, recruitment‐group effects and random‐intercept heterogeneity should not be interpreted as pure between‐hospital effects. Generalisability to smaller, rural, nontertiary and non‐Chinese operating‐room settings is therefore uncertain. The relative ranking of support priorities may also vary across institutional and national settings. A conventional response rate could not be calculated, and selection, participation‐pressure and administration‐mode biases cannot be excluded. Finally, this survey measured self‐reported work scenarios rather than environmental or biological microplastic exposure, and the open‐ended component was based on a small self‐selected subset. Future studies should combine validated scenario measures with objective exposure assessment and broader external validation.

## 7. Conclusion

In this multicentre survey of operating‐room nursing staff, night‐shift work was associated with higher self‐reported occupational scenario burden, with positive point estimates across the principal sensitivity analyses, whereas the orthopaedic association was partly dependent on specialty‐specific index content. Staff prioritised several organisational responses, including rest‐area infrastructure, meal‐container reform, ventilation and smoke management, occupational‐health monitoring, nonplastic alternatives and night‐shift support. These findings provide a work‐system agenda for nursing management and a basis for prospective studies integrating validated scenario measures with objective environmental and biological assessment.

## Author Contributions

Jie Yang, Panyu Chen, Zhihui Huang, Lin Yang: conceptualisation, methodology, investigation, data curation, formal analysis, writing–original draft.

Qian Feng, Qinghua Zhang, Jun Cheng, Qihao Chen: methodology, investigation, data curation, writing–review and editing.

Xiaoli Zhong, Junhong Zhou, Meng Shi: investigation, resources, writing–review and editing.

Yongqiong Tan: supervision, project administration, writing–review and editing.

Yonghua Chen: conceptualisation, methodology, supervision, project administration, funding acquisition, writing–review and editing.

## Funding

This work was supported by the Regional Collaborative Innovation Project of Sichuan Provincial Department of Science and Technology (Grant No. 2026YFHZ0184) and the Scientific Research Incubation Fund, West China Tianfu Hospital, Sichuan University (Grant No. TFFHZ20251002).

## Disclosure

The funders had no role in the study design, data collection, data analysis, data interpretation, writing of the report or decision to submit the article for publication. All authors reviewed and approved the final version of the manuscript.

## Ethics Statement

The Medical Ethics Review Committee of West China Tianfu Hospital, Sichuan University, approved the study on 4 November 2025 (No. 2025‐066). Participants received information about the study purpose, voluntary participation, confidentiality and intended data use before providing written or electronic informed consent. Information used for duplicate checking was retained only for data‐quality verification and removed before analysis; the analytic dataset was de‐identified before analysis and reporting.

## Conflicts of Interest

The authors declare no conflicts of interest.

## Supporting Information

Additional supporting information can be found online in the Supporting Information section.

## Supporting information


**Supporting Information** The supporting information includes Supporting Tables S1–S17 and Supporting Figure S1, which provide additional details on the questionnaire‐derived scenario‐burden measures and scoring, recruitment‐group characteristics, questionnaire completeness, sensitivity and robustness analyses and regression diagnostics. Supporting File 1 contains the anonymised Chinese questionnaire used for data collection, and Supporting File 2 contains the English reporting translation, which was not used for data collection.

## Data Availability

The data that support the findings of this study are available from the corresponding author upon reasonable request.
