# Supplementary material for: Self‐Reported Plastic‐Related Occupational Scenarios and Perceived Support Priorities Among Operating‐Room Nursing Staff: A Multicentre Cross‐Sectional Survey
Source: J Nurs Manag. 2026 Oct 17;2026:7475740. doi: 10.1155/jonm/7475740 (PMC13675910; doi:10.1155/jonm/7475740)
Supplement: Supplementary file 1 [file JONM-2026-7475740-s001.zip › STROBE_checklist_cross-sectional_JNM_submission_UPDATED_20260928.pdf]

STROBE checklist - cross-sectional studies

Item	No.	Recommendation	Page No / details
Title and abstract	1a	Indicate the study design with a commonly used term in the title or abstract.	p. 1
Title and abstract	1b	Provide in the abstract an informative and balanced summary of what was done and what was found.	p. 4
Background/rationale	2	Explain the scientific background and rationale for the investigation.	pp. 4–5
Objectives	3	State specific objectives, including any prespecified hypotheses.	p. 5
Study design	4	Present key elements of study design early in the paper.	p. 5
Setting	5	Describe setting, locations and relevant dates, including data-collection period.	pp. 5–6
Participants	6a	Give eligibility criteria and sources/methods of selection of participants.	pp. 5–6 and p. 9; Figure 2 (legend p. 20)
Variables	7	Define outcomes, exposures, predictors, potential confounders and effect modifiers.	pp. 6–9; Figure 1 (legend p. 20); Supplementary Tables S1–S2 (pp. 2–4)
Data sources/measurement	8*	Give sources of data and measurement methods for variables of interest.	pp. 5–8; Supplementary Files 1–2; Supplementary Tables S1–S5 (pp. 2–6)
Bias	9	Describe efforts to address potential sources of bias.	pp. 5–6, 8–9 and 12–14; Supplementary Tables S5, S13–S15 and S17
Study size	10	Explain how study size was arrived at.	p. 6. Recruitment-yield approach: all eligible submissions during the predefined collection period were included.
Quantitative variables	11	Explain how quantitative variables were handled and grouped.	pp. 7–8; Supplementary Table S1 (pp. 2–4) and Supplementary Table S11 (pp. 13–14)
Statistical methods	12a	Describe statistical methods, including control for confounding.	pp. 8–9
Statistical methods	12b	Describe subgroup/interaction methods.	No interaction analyses were performed. Exploratory analyses restricted to participants reporting orthopaedic work are reported in Supplementary Table S9 (p. 10).
Statistical methods	12c	Explain how missing data were addressed.	pp. 6–9; Supplementary Table S5 (pp. 6–7)
Statistical methods	12d	Describe methods accounting for sampling strategy, if applicable.	pp. 5 and 8–9. Convenience sampling was used; recruitment-group fixed effects adjusted for recruitment context, with random-intercept and leave-one-group-out sensitivity analyses in Supplementary Table S14 (p. 17).
Statistical methods	12e	Describe sensitivity analyses.	pp. 8–11; Supplementary Tables S8–S15 and S17
Participants	13a	Report numbers at each stage of the study.	p. 9; Figure 2 (legend p. 20)

Item	No.	Recommendation	Page No / details
Participants	13b	Give reasons for non-participation at each stage.	pp. 5–6, p. 9 and p. 14; Figure 2 (legend p. 20). A unique invited/eligible denominator and non-response counts were unavailable.
Participants	13c	Consider use of a flow diagram.	Figure 2 (legend p. 20)
Descriptive data	14a	Give characteristics of participants and information on exposures/potential confounders.	p. 9; Table 1 (p. 17); Supplementary Tables S2–S4 (pp. 4–5)
Descriptive data	14b	Indicate number of participants with missing data for each variable of interest.	pp. 6–7 and p. 9; Supplementary Table S5 (pp. 6–7)
Outcome data	15*	Report numbers of outcome events or summary measures.	pp. 9–11; Tables 1–3 (pp. 17–19)
Main results	16a	Give unadjusted/adjusted estimates and precision; clarify adjusted confounders.	pp. 10–11; Table 2 (p. 18); Supplementary Tables S7–S15
Main results	16b	Report category boundaries when continuous variables were categorised.	p. 8; Supplementary Table S11 (pp. 13–14), Supplementary Table S12 (p. 15), and Supplementary Figure S1 (p. 22)
Main results	16c	Translate relative risk into absolute risk for meaningful period, if relevant.	No follow-up risk period applies in this cross-sectional study. For the exploratory binary outcome, model-standardised probabilities and absolute probability differences are reported on p. 11 and in Supplementary Table S12 (p. 15).
Other analyses	17	Report other analyses, including sensitivity analyses.	pp. 10–11; Supplementary Tables S8–S17
Key results	18	Summarise key results with reference to study objectives.	p. 12
Limitations	19	Discuss limitations and potential bias/imprecision.	p. 14
Interpretation	20	Give cautious overall interpretation considering objectives, limitations and evidence.	pp. 12–14
Generalisability	21	Discuss generalisability/external validity.	p. 14
Funding	22	Give funding source and role of funders.	Title page, p. 2, Funding statement
