# Supplementary material for: Self‐Reported Plastic‐Related Occupational Scenarios and Perceived Support Priorities Among Operating‐Room Nursing Staff: A Multicentre Cross‐Sectional Survey
Source: J Nurs Manag. 2026 Oct 17;2026:7475740. doi: 10.1155/jonm/7475740 (PMC13675910; doi:10.1155/jonm/7475740)
Supplement: Supplementary file 1 [file JONM-2026-7475740-s001.zip › JNM_Supplementary_Materials_20260927.docx]

**Supplementary Materials**

**Self-reported plastic-related occupational scenarios and perceived support priorities among operating-room nursing staff: a multicentre cross-sectional survey**

**Contents**
Supplementary Tables S1–S17
Supplementary Figure S1
Supplementary Files 1–2: Chinese questionnaire and English reporting translation

**Supplementary Table S1. Conceptual map and scoring framework for questionnaire-derived scenario-burden indices**

**Panel A. Occupational scenario-burden items: conceptual role, potential pathway, scoring, and limitations**

| **Item** | **Scenario** | **Conceptual domain** | **Potential material/pathway represented** | **Primary context captured** | **Score** | **Applicability / key limitation** |
| --- | --- | --- | --- | --- | --- | --- |
| C4.1 | Medical plastic-item handling | General material handling | Direct handling of plastic-containing medical consumables, packaging, or disposable materials | Material-contact / task-frequency context | 0-4 | Broadly applicable; handling frequency is not a particle-dose measure and material composition was not individually verified. |
| C4.2 | Disposable gown duration | Apparel / PPE | Duration of contact with disposable surgical gowns; potential synthetic/polymer-containing textile context | Material-contact duration | 0-4 | Composition varies by product; duration does not quantify particle release or inhalation. |
| C4.3 | Disposable cap use | Apparel / PPE | Frequency of disposable surgical-cap use; potential synthetic/polymer-containing textile context | Material-use frequency | 0-4 | High prevalence and limited between-person contrast; product composition not verified. |
| C4.4 | Sterile-package opening | Packaging / material handling | Repeated opening of sterile or disposable packaging, potentially including polymer-containing packaging | Packaging-handling / task-intensity context | 0-3 | Unable-to-estimate responses are non-scorable; packaging composition and particle generation were not measured. |
| C4.5 | Surgical draping | Draping / material handling | Handling or placement of disposable surgical drapes, which may contain synthetic/polymer components | Material-handling / procedural-volume context | 0-4 | Partly reflects case volume and task assignment; material composition not verified. |
| C4.6 | PVC glove use | Polymer material use | Use of polyvinyl chloride gloves | Direct polymer-material use | 0-3 | Counts material use, not environmental or biological exposure. |
| C4.7 | Surgical smoke occurrence | Smoke / airborne particulate context | Visible or noticeable surgical smoke during relevant procedures | Airborne smoke/particulate context | 0-3 | Not plastic-specific; smoke may contain heterogeneous particulate and chemical constituents. |
| C4.8 | Immediate smoke evacuation in open surgery | Environmental control / smoke management | Availability and timely use of smoke-evacuation equipment during open procedures | Environmental-control context (reverse control coding) | 0-4 | Captures control practice rather than material contact; not specific to plastic or microplastic particles. |
| C4.9 | Laparoscopic smoke management | Environmental control / smoke management | Active evacuation versus passive release of laparoscopic smoke | Airborne-release / control context | 0,1,2,4 | Not plastic-specific; “not involved/no fixed pattern” is non-scorable; non-consecutive coding reflects the administered response-option structure. |
| C4.10 | Bone cement or pulse-lavage work | Specialty-specific procedural context | Participation in procedures involving bone cement and/or pulse lavage | Polymer/chemical and aerosol-generating procedural context | 0-4 | Strongly specialty-specific; combines distinct processes and overlaps with orthopaedic work, creating potential circularity in orthopaedic analyses. |

Interpretive note. The occupational items were retained as a formative scenario profile, not as biologically equivalent exposure events. The conceptual map distinguishes plastic/material-contact contexts from smoke/particulate and environmental-control contexts and identifies the specialty-specific nature of C4.10. Abbreviations: PPE, personal protective equipment; PVC, polyvinyl chloride.

**Panel B. Occupational item response-option scoring map**

| **Item** | **Response option → analysis score** | **Non-scorable handling** | **Max** |
| --- | --- | --- | --- |
| C4.1 | Very little contact=0; Moderate (<10/day)=1; High (<20/day)=2; Frequent (20–30/day)=3; Very frequent (>30/day)=4 | None | 4 |
| C4.2 | Almost never=0; <2 h=1; 2–4 h=2; 4–6 h=3; >6 h=4 | None | 4 |
| C4.3 | Almost never=0; Occasionally=1; Sometimes=2; Often=3; Always=4 | None | 4 |
| C4.4 | Few (1–30)=0; Many (31–60)=1; Very many (61–90)=2; Extremely many (>90)=3 | Unfixed/highly variable/difficult to estimate → non-scorable | 3 |
| C4.5 | Not involved=0; Few (1–2 operations)=1; Moderate (3–5)=2; Many (5–9)=3; Extremely many (≥10)=4 | None; 'Not involved' is a valid zero-score category | 4 |
| C4.6 | 0 pairs=0; 1–2 pairs=1; 3–4 pairs=2; ≥5 pairs=3 | None | 3 |
| C4.7 | Not involved in such operations=0; Almost never=0; Occasionally (<10%)=1; Sometimes (10%–50%)=2; Often (≥50%)=3 | None; 'Not involved' is a valid zero-score category | 3 |
| C4.8 | Always immediately available=0; Often=1; About half=2; Rarely=3; Almost never used/equipment response not timely=4 | None; higher scores indicate less immediate smoke-control availability/use | 4 |
| C4.9 | Routine active protection=0; Frequent active protection=1; Mixed use=2; Routine passive release=4 | Not involved/no fixed mode → non-scorable; score 3 was not used by the questionnaire | 4 |
| C4.10 | Almost not involved=0; Occasionally (a few times/month)=1; Once/week=2; 2–4 times/week=3; ≥1/day=4 | None; 'Almost not involved' is a valid zero-score category | 4 |

Scoring note. The response-option mapping above reproduces the response categories as administered and their analysis coding. Scores represent ordinal work-scenario intensity/control coding for index construction and are not biological potency weights. Designed non-scorable categories were excluded from the person-specific denominator; valid zero-score categories remained in the denominator. Where original category wording was overlapping or non-consecutive, the respondent's selected category was used as recorded and was not retrospectively reassigned.

**Panel C. Daily-life scenario-burden items and original scoring**

| **Item** | **Scenario** | **Assigned score** | **Maximum** |
| --- | --- | --- | --- |
| C1.2 | Drinking container at work | Stainless steel/glass/ceramic=0; variable=2; disposable paper=3; plastic=4 | 4 |
| C1.3 | Drinking container outside work | Stainless steel/glass/ceramic=0; variable=2; disposable paper=3; plastic=4 | 4 |
| C1.4 | Bottled drinks at work | Never=0; <1/week=1; 2-3/week=2; once/day=3; multiple/day=4 | 4 |
| C1.5 | Bottled drinks outside work | Never=0; <1/week=1; 2-3/week=2; once/day=3; multiple/day=4 | 4 |
| C1.6 | Disposable paper cups at work | Never=0; rarely=1; about half=2; often=3; almost always=4 | 4 |
| C1.7 | Disposable paper cups outside work | Never=0; rarely=1; about half=2; often=3; almost always=4 | 4 |
| C2.1 | Hot food in plastic containers/bags at work | Never=0; <30%=1; 30-50%=2; 50-80%=3; >80%=4 | 4 |
| C2.2 | Hot food in plastic containers/bags outside work | Never=0; <30%=1; 30-50%=2; 50-80%=3; >80%=4 | 4 |
| C3.1 | Toothpaste and microbeads | Deliberately avoids=0; no fixed choice=2; unknown content=3; known use=4 | 4 |
| C3.2 | Exfoliating scrub use | Never=0; several/month=1; 1-2/week=2; 3-5/week=3; daily=4 | 4 |

**Panel D. Index calculation and alternative occupational-score definitions used for sensitivity analyses**

| **Code** | **Definition** | **Items / calculation** | **Completeness condition** | **Interpretive role** |
| --- | --- | --- | --- | --- |
| O10 | Original 10-item occupational index | C4.1-C4.10; person-specific valid-item denominator | Observed: 8–10 valid items; no lower count occurred | Principal continuous outcome. |
| B9 | Bone-cement/pulse-lavage-excluded index | O10 excluding C4.10 | At least 8/9 valid items | Primary circularity sensitivity for orthopaedic involvement. |
| G6 | Six-item general/common-scenario index | C4.1-C4.6; all six complete; fixed maximum 22 | 6/6 valid | Reduces specialty-specific and smoke-management content; “common” denotes broader applicability, not proven identical opportunity across jobs. |
| GE6 | Equal-item-weighted six-item general/common index | C4.1–C4.6; each item divided by its fixed item maximum, then averaged ×100 | 6/6 valid | Tests equal item contribution within the complete six-item common/general subset; used in sensitivity and boundary diagnostics. |
| G6A | Six-item general/common index allowing one missing item | C4.1-C4.6; person-specific valid-item maximum | At least 5/6 valid | Assesses sensitivity to complete-item restriction. |
| E10 | Equal-item-weighted 10-item index | Each C4 item divided by its fixed item maximum, then averaged x100 | At least 8/10 valid | Tests whether unequal item score ranges drive results. |
| N8 | Reduced 8-item index | C4.1-C4.3, C4.5-C4.8, C4.10; excludes C4.4 and C4.9 | At least 7/8 valid | Alternative scoring sensitivity; retains C4.10 and therefore does not resolve orthopaedic circularity. |
| S3 | Smoke-related subdomain | C4.7-C4.9 complete; sum/11 x100 | 3/3 valid | Exploratory smoke/airborne-management domain. |
| B1 | Bone cement/pulse-lavage single-item score | C4.10/4 x100 | C4.10 valid | Descriptive specialty-specific task score; not an independent validation measure. |

Scoring formula for O10 and analogous person-specific-denominator indices: score = (sum of observed item scores / sum of corresponding item maxima) × 100. When all occupational items were observed, the maximum raw total was 37; for the daily-life index, 40.

**Supplementary Table S2. Cognitive-behavioural construct composition, scoring, reliability, and post hoc refinements**

| **Measure** | **Items** | **Scale** | **Mean (SD), n=415** | **Raw α** | **Standardised α** | **Average inter-item r** | **Analysis/refinement note** |
| --- | --- | --- | --- | --- | --- | --- | --- |
| Self-perceived awareness | 1 | 1–10 | 5.9 (2.4) | NA | NA | NA | Single-item rating |
| Concern | 1 | 1–10 | 6.0 (2.5) | NA | NA | NA | Single-item rating |
| Attitude | 3 | 1–10 | 8.1 (1.6) | 0.829 | 0.835 | 0.628 | Post hoc 3-item score after removing reverse-worded health-dismissal item |
| Norm pressure | 3 | 1–10 | 7.1 (1.8) | 0.762 | 0.761 | 0.516 | Post hoc 3-item score after separating peer environmental influence |
| Peer environmental influence | 1 | 1–10 | 7.3 (2.0) | NA | NA | NA | Analysed separately |
| Perceived behavioural control | 4 | 1–10 | 7.5 (1.8) | 0.877 | 0.877 | 0.641 | No post hoc item deletion reported |
| Behavioural intention | 4 | 1–10 | 7.7 (1.8) | 0.930 | 0.931 | 0.770 | No post hoc item deletion reported |
| Protective behaviour | 5 | 0–100 | 59.4 (25.3) | 0.882 | 0.883 | 0.602 | No post hoc item deletion reported |

SD, standard deviation; NA, not applicable. The occupational and daily-life scenario-burden indices were formative measures and were not evaluated with Cronbach's alpha.

The attitude and norm-pressure refinements were analysis-stage, post hoc decisions and were not prospectively prespecified.

**Supplementary Table S3. Recruitment-group contribution, survey mode, and analytical sample counts**

| **Recruitment group** | **Type** | **Broad geography** | **Mode** | **Eligible n** | **Frontline n** | **Complete-case n** | **O10 mean (SD)** | **Upper-tertile events (complete-case n=408)** |
| --- | --- | --- | --- | --- | --- | --- | --- | --- |
| Group A | Hospital-based | Southwestern China | Online | 121 | 118 | 117 | 47.1 (12.4) | 31 |
| Group B | Hospital-based | Southwestern China | Online | 76 | 66 | 65 | 48.6 (9.2) | 11 |
| Group C/J | Visiting-nurse cohort | Multiple home institutions | Online | 84 | 68 | 65 | 51.9 (14.1) | 23 |
| Group D | Hospital-based | Southwestern China | Paper | 44 | 40 | 39 | 55.9 (9.7) | 22 |
| Group E | Hospital-based | Southwestern China | Online | 42 | 42 | 42 | 53.1 (10.9) | 17 |
| Group F | Hospital-based | Southwestern China | Paper | 29 | 29 | 29 | 55.9 (13.7) | 11 |
| Group G | Hospital-based | Southeastern China | Online | 19 | 19 | 19 | 44.1 (14.0) | 4 |
| Group H | Hospital-based | Southwestern China | Online | 18 | 18 | 17 | 59.1 (10.0) | 11 |
| Group I | Hospital-based | Southwestern China | Online | 15 | 15 | 15 | 51.2 (10.4) | 5 |

O10, original 10-item occupational scenario-burden index; SD, standard deviation. Upper-tertile events are shown only for sparse-cell assessment of the secondary binary model.

Group C/J represents one visiting-nurse training/recruitment cohort comprising nurses from multiple home institutions; recruitment-group variation therefore reflects recruitment context and should not be interpreted as pure between-hospital variation.

**Supplementary Table S4. Role and surgical-specialty composition among frontline participants by recruitment group**

| **Group** | **n** | **Scrub/instrument** | **Circulating** | **Anaesthesia monitoring** | **Management/quality** | **General surgery** | **Orthopaedics** | **Interventional** | **Gynaecology/urology** |
| --- | --- | --- | --- | --- | --- | --- | --- | --- | --- |
| Group A | 118 | 68.6% | 61.9% | 0.8% | 5.1% | 50.0% | 20.3% | 12.7% | 29.7% |
| Group B | 66 | 50.0% | 62.1% | 6.1% | 6.1% | 60.6% | 31.8% | 16.7% | 33.3% |
| Group C/J | 68 | 73.5% | 70.6% | 4.4% | 17.6% | 64.7% | 67.6% | 10.3% | 69.1% |
| Group D | 40 | 77.5% | 92.5% | 0.0% | 7.5% | 32.5% | 22.5% | 0.0% | 95.0% |
| Group E | 42 | 69.0% | 76.2% | 0.0% | 0.0% | 35.7% | 50.0% | 2.4% | 50.0% |
| Group F | 29 | 69.0% | 72.4% | 0.0% | 0.0% | 89.7% | 55.2% | 20.7% | 82.8% |
| Group G | 19 | 100.0% | 73.7% | 0.0% | 10.5% | 89.5% | 47.4% | 26.3% | 63.2% |
| Group H | 18 | 72.2% | 61.1% | 0.0% | 0.0% | 38.9% | 38.9% | 5.6% | 22.2% |
| Group I | 15 | 53.3% | 60.0% | 13.3% | 0.0% | 80.0% | 53.3% | 0.0% | 60.0% |

Roles and surgical specialties were multiple-response items; percentages within a recruitment group therefore do not sum to 100%.

**Supplementary Table S5. Item-level non-scorable responses and valid-item denominators for the occupational scenario-burden index**

**Panel A. Item-level non-scorable responses and valid-score counts**

| **Item** | **Scenario** | **Valid score n** | **Non-scorable n (%)** | **Zero-score n (%)** |
| --- | --- | --- | --- | --- |
| C4.1 | Medical plastic-item handling | 415 | 0 (0.0) | 25 (6.0) |
| C4.2 | Disposable gown duration | 415 | 0 (0.0) | 139 (33.5) |
| C4.3 | Disposable cap use | 415 | 0 (0.0) | 47 (11.3) |
| C4.4 | Sterile-package opening | 365 | 50 (12.0) | 70 (16.9) |
| C4.5 | Surgical draping | 415 | 0 (0.0) | 21 (5.1) |
| C4.6 | PVC glove use | 415 | 0 (0.0) | 3 (0.7) |
| C4.7 | Surgical smoke occurrence | 415 | 0 (0.0) | 66 (15.9) |
| C4.8 | Immediate smoke evacuation | 415 | 0 (0.0) | 82 (19.8) |
| C4.9 | Laparoscopic smoke management | 386 | 29 (7.0) | 124 (29.9) |
| C4.10 | Bone cement/pulse-lavage work | 415 | 0 (0.0) | 173 (41.7) |

“Non-scorable” includes questionnaire responses that could not be assigned an analysis score (for example, unable-to-estimate or no-fixed-pattern responses); it should not be interpreted uniformly as item nonresponse or non-applicability.

A zero score represents the lowest coded category and was not automatically reclassified as non-applicable.

**Panel B. Number of valid items contributing to the original 10-item occupational index (O10)**

| **Valid items** | **n** | **% of n=415** |
| --- | --- | --- |
| 10 | 345 | 83.1% |
| 9 | 61 | 14.7% |
| 8 | 9 | 2.2% |

All 415 frontline participants had at least 8 of 10 valid occupational items and therefore a calculable O10 score.

**Panel C. O10 valid-item counts by recruitment group**

| **Group** | **N** | **10 valid** | **9 valid** | **8 valid** |
| --- | --- | --- | --- | --- |
| Group A | 118 | 105 (89.0) | 12 (10.2) | 1 (0.8) |
| Group B | 66 | 53 (80.3) | 11 (16.7) | 2 (3.0) |
| Group C/J | 68 | 56 (82.4) | 10 (14.7) | 2 (2.9) |
| Group D | 40 | 28 (70.0) | 8 (20.0) | 4 (10.0) |
| Group E | 42 | 35 (83.3) | 7 (16.7) | 0 (0.0) |
| Group F | 29 | 25 (86.2) | 4 (13.8) | 0 (0.0) |
| Group G | 19 | 16 (84.2) | 3 (15.8) | 0 (0.0) |
| Group H | 18 | 14 (77.8) | 4 (22.2) | 0 (0.0) |
| Group I | 15 | 13 (86.7) | 2 (13.3) | 0 (0.0) |

**Panel D. O10 valid-item counts by selected role and specialty**

| **Stratum** | **Category** | **N** | **10 valid** | **9 valid** | **8 valid** |
| --- | --- | --- | --- | --- | --- |
| Scrub | Yes | 284 | 237 (83.5) | 42 (14.8) | 5 (1.8) |
| Scrub | No | 131 | 108 (82.4) | 19 (14.5) | 4 (3.1) |
| Circulating | Yes | 286 | 233 (81.5) | 46 (16.1) | 7 (2.4) |
| Circulating | No | 129 | 112 (86.8) | 15 (11.6) | 2 (1.6) |
| Anaesthesia monitoring | Yes | 10 | 10 (100.0) | 0 (0.0) | 0 (0.0) |
| Anaesthesia monitoring | No | 405 | 335 (82.7) | 61 (15.1) | 9 (2.2) |
| Orthopaedic | Yes | 161 | 135 (83.9) | 25 (15.5) | 1 (0.6) |
| Orthopaedic | No | 254 | 210 (82.7) | 36 (14.2) | 8 (3.1) |
| Interventional | Yes | 46 | 37 (80.4) | 8 (17.4) | 1 (2.2) |
| Interventional | No | 369 | 308 (83.5) | 53 (14.4) | 8 (2.2) |

These comparisons describe score-completeness patterns only; they do not establish equal opportunity for each occupational scenario across roles or specialties.

**Supplementary Table S6. Selected self-reported daily-life and occupational scenarios among frontline operating-room nursing staff**

| **Scenario** | **Overall n=415** | **Lower two tertiles n=277** | **Upper tertile n=138** | **Descriptive P** |
| --- | --- | --- | --- | --- |
| Work bottled water/drinks at least daily | 102 (24.6) | 67 (24.2) | 35 (25.4) | 0.888 |
| Out-of-work bottled water/drinks at least daily | 79 (19.0) | 50 (18.1) | 29 (21.0) | 0.554 |
| Work hot food in plastic containers/bags at least 50% of the time | 153 (36.9) | 94 (33.9) | 59 (42.8) | 0.100 |
| Out-of-work hot food in plastic containers/bags at least 50% of the time | 95 (22.9) | 62 (22.4) | 33 (23.9) | 0.822 |
| Plastic medical-item handling ≥20 times/day | 134 (32.3) | 58 (20.9) | 76 (55.1) | <0.001 |
| Disposable surgical gown use ≥4 h/day shift | 100 (24.1) | 40 (14.4) | 60 (43.5) | <0.001 |
| Surgical cap worn always or often | 285 (68.7) | 174 (62.8) | 111 (80.4) | <0.001 |
| Disposable sterile packages opened >60/day shift | 201 (48.4) | 101 (36.5) | 100 (72.5) | <0.001 |
| Surgical draping ≥5 operations/day shift | 152 (36.6) | 72 (26.0) | 80 (58.0) | <0.001 |
| PVC gloves ≥3 pairs/day shift | 379 (91.3) | 243 (87.7) | 136 (98.6) | <0.001 |
| Visible/odorous smoke in ≥10% of relevant operations | 288 (69.4) | 170 (61.4) | 118 (85.5) | <0.001 |
| Open-surgery smoke evacuation not always immediately available/used | 333 (80.2) | 214 (77.3) | 119 (86.2) | 0.042 |
| Laparoscopic smoke sometimes/directly released into room air | 146 (35.2) | 79 (28.5) | 67 (48.6) | <0.001 |
| Bone cement/pulse-lavage work at least weekly | 119 (28.7) | 56 (20.2) | 63 (45.7) | <0.001 |

Data are n (%). Scenario variables were dichotomised from the original ordinal questionnaire items for descriptive presentation only. PVC, polyvinyl chloride.

The occupational scenario items contribute directly to the occupational score used to form the tertile groups; these between-group contrasts therefore cannot independently validate or corroborate the index.

P values are descriptive and not confirmatory.

**Supplementary Table S7. Full principal continuous regression models for self-reported occupational scenario burden**

**Panel A. Model 1 (n=408)**

| **Associated factor** | **β (classical 95% CI)** | **P** | **β (HC3 95% CI)** | **P** |
| --- | --- | --- | --- | --- |
| Age, per year | 0.00 (-0.32 to 0.31) | 0.983 | 0.00 (-0.31 to 0.30) | 0.982 |
| Male sex | 2.77 (-0.51 to 6.05) | 0.098 | 2.77 (-0.39 to 5.93) | 0.085 |
| Recruitment group: B vs A | 1.45 (-2.37 to 5.26) | 0.456 | 1.45 (-2.12 to 5.02) | 0.426 |
| Recruitment group: C/J vs A | 3.89 (-0.27 to 8.06) | 0.067 | 3.89 (-0.92 to 8.70) | 0.112 |
| Recruitment group: D vs A | 9.17 (4.85 to 13.48) | <0.001 | 9.17 (5.22 to 13.11) | <0.001 |
| Recruitment group: E vs A | 4.98 (0.65 to 9.30) | 0.024 | 4.98 (0.73 to 9.22) | 0.022 |
| Recruitment group: F vs A | 7.18 (2.33 to 12.04) | 0.004 | 7.18 (1.57 to 12.79) | 0.012 |
| Recruitment group: G vs A | -5.06 (-10.88 to 0.75) | 0.088 | -5.06 (-11.91 to 1.79) | 0.147 |
| Recruitment group: H vs A | 9.90 (3.93 to 15.86) | 0.001 | 9.90 (4.45 to 15.34) | <0.001 |
| Recruitment group: I vs A | 2.86 (-3.62 to 9.34) | 0.386 | 2.86 (-3.23 to 8.95) | 0.356 |
| Operating-room work years, per ordinal category | -0.40 (-1.80 to 1.00) | 0.576 | -0.40 (-1.81 to 1.02) | 0.581 |
| Any night shift | 3.86 (0.99 to 6.73) | 0.008 | 3.86 (0.96 to 6.76) | 0.009 |
| Participation in orthopaedic surgery | 4.93 (2.36 to 7.50) | <0.001 | 4.93 (2.42 to 7.44) | <0.001 |
| Participation in interventional surgery | 1.94 (-1.92 to 5.79) | 0.324 | 1.94 (-1.61 to 5.49) | 0.284 |

Group A is the recruitment-group reference category. Recruitment-group coefficients are nuisance adjustment terms and are not institutional causal comparisons.

**Panel B. Model 2 (n=408)**

| **Associated factor** | **β (classical 95% CI)** | **P** | **β (HC3 95% CI)** | **P** |
| --- | --- | --- | --- | --- |
| Age, per year | 0.00 (-0.31 to 0.31) | 0.999 | 0.00 (-0.30 to 0.30) | 0.999 |
| Male sex | 2.24 (-1.09 to 5.56) | 0.187 | 2.24 (-0.96 to 5.43) | 0.170 |
| Recruitment group: B vs A | 0.90 (-2.95 to 4.76) | 0.646 | 0.90 (-2.78 to 4.59) | 0.631 |
| Recruitment group: C/J vs A | 2.96 (-1.25 to 7.17) | 0.168 | 2.96 (-1.93 to 7.85) | 0.235 |
| Recruitment group: D vs A | 8.83 (4.36 to 13.30) | <0.001 | 8.83 (4.35 to 13.31) | <0.001 |
| Recruitment group: E vs A | 4.16 (-0.14 to 8.47) | 0.058 | 4.16 (-0.27 to 8.59) | 0.065 |
| Recruitment group: F vs A | 6.34 (1.33 to 11.34) | 0.013 | 6.34 (0.63 to 12.05) | 0.030 |
| Recruitment group: G vs A | -4.61 (-10.44 to 1.21) | 0.120 | -4.61 (-11.49 to 2.26) | 0.187 |
| Recruitment group: H vs A | 9.80 (3.89 to 15.70) | 0.001 | 9.80 (4.37 to 15.22) | <0.001 |
| Recruitment group: I vs A | 1.69 (-4.80 to 8.17) | 0.609 | 1.69 (-4.52 to 7.89) | 0.593 |
| Operating-room work years, per ordinal category | -0.22 (-1.62 to 1.18) | 0.760 | -0.22 (-1.66 to 1.23) | 0.767 |
| Any night shift | 3.17 (0.26 to 6.08) | 0.033 | 3.17 (0.22 to 6.12) | 0.035 |
| Participation in orthopaedic surgery | 4.90 (2.35 to 7.45) | <0.001 | 4.90 (2.40 to 7.41) | <0.001 |
| Participation in interventional surgery | 1.75 (-2.08 to 5.58) | 0.370 | 1.75 (-1.77 to 5.27) | 0.330 |
| Daily-life scenario-burden index, per 1 SD | 0.71 (-0.48 to 1.90) | 0.243 | 0.71 (-0.50 to 1.92) | 0.251 |
| Concern score, per 1 SD | 1.41 (0.02 to 2.79) | 0.046 | 1.41 (-0.07 to 2.88) | 0.062 |
| Attitude score, per 1 SD | 2.08 (0.54 to 3.61) | 0.008 | 2.08 (-0.22 to 4.38) | 0.077 |
| Norm pressure score, per 1 SD | 0.82 (-0.56 to 2.20) | 0.245 | 0.82 (-0.75 to 2.40) | 0.306 |
| Peer environmental influence, per 1 SD | -1.09 (-2.70 to 0.52) | 0.183 | -1.09 (-2.81 to 0.63) | 0.213 |
| Perceived behavioural control, per 1 SD | -0.86 (-2.91 to 1.18) | 0.408 | -0.86 (-3.02 to 1.30) | 0.433 |
| Behavioural intention, per 1 SD | -1.91 (-3.98 to 0.16) | 0.071 | -1.91 (-4.32 to 0.50) | 0.119 |
| Protective behaviour, per 1 SD | 0.11 (-1.16 to 1.38) | 0.870 | 0.11 (-1.26 to 1.47) | 0.879 |

Continuous daily-life and cognitive-behavioural covariates were standardised to 1 SD. Model 2 is an extended exploratory model, not a superior causal-adjustment model. Abbreviations: CI, confidence interval; HC3, heteroskedasticity-consistent type 3; SD, standard deviation.

**Supplementary Table S8. Alternative index definitions and orthopaedic-circularity sensitivity analyses**

**Panel A. Model 1 key effects across alternative continuous index definitions**

| **Index** | **Definition** | **Model n** | **Night-shift β (HC3 95% CI)** | **P** | **Orthopaedic β (HC3 95% CI)** | **P** |
| --- | --- | --- | --- | --- | --- | --- |
| O10 | Original 10-item index | 408 | 3.86 (0.96 to 6.76) | 0.009 | 4.93 (2.42 to 7.44) | <0.001 |
| B9 | Exclude C4.10 bone cement/pulse lavage | 399 | 4.34 (1.27 to 7.41) | 0.006 | 2.47 (-0.14 to 5.09) | 0.064 |
| G6 | Six general/common items, complete | 358 | 4.14 (-0.01 to 8.28) | 0.051 | 1.83 (-1.65 to 5.32) | 0.302 |
| G6A | Six general/common items, allow one missing | 408 | 5.41 (1.69 to 9.13) | 0.004 | 2.49 (-0.81 to 5.78) | 0.139 |
| E10 | Equal-item-weighted 10-item index | 408 | 3.98 (1.02 to 6.95) | 0.009 | 4.93 (2.41 to 7.45) | <0.001 |
| N8 | Exclude C4.4/C4.9; retain C4.10 | 408 | 3.94 (1.03 to 6.86) | 0.008 | 5.28 (2.79 to 7.76) | <0.001 |

All models included the same Model 1 covariates and recruitment-group fixed effects.

E10 tests numerical weighting. B9 and G6 directly address specialty-specific content. N8 retains C4.10 and therefore does not resolve orthopaedic circularity.

**Panel B. Same-sample comparisons with the original O10 score**

| **Alternative index** | **Factor** | **Same n** | **O10 β (HC3 95% CI)** | **Alternative β (HC3 95% CI)** | **O10−alternative β** | **Descriptive change** |
| --- | --- | --- | --- | --- | --- | --- |
| B9 | Night shift | 399 | 3.82 (0.88 to 6.76) | 4.34 (1.27 to 7.41) | -0.52 | -13.6% |
| B9 | Orthopaedic involvement | 399 | 5.01 (2.48 to 7.54) | 2.47 (-0.14 to 5.09) | 2.54 | 50.7% |
| G6 | Night shift | 358 | 3.00 (-0.16 to 6.15) | 4.14 (-0.01 to 8.28) | -1.14 | -38.1% |
| G6 | Orthopaedic involvement | 358 | 4.74 (2.06 to 7.42) | 1.83 (-1.65 to 5.32) | 2.91 | 61.4% |
| G6A | Night shift | 408 | 3.86 (0.96 to 6.76) | 5.41 (1.69 to 9.13) | -1.55 | -40.1% |
| G6A | Orthopaedic involvement | 408 | 4.93 (2.42 to 7.44) | 2.49 (-0.81 to 5.78) | 2.44 | 49.6% |
| E10 | Night shift | 408 | 3.86 (0.96 to 6.76) | 3.98 (1.02 to 6.95) | -0.12 | -3.2% |
| E10 | Orthopaedic involvement | 408 | 4.93 (2.42 to 7.44) | 4.93 (2.41 to 7.45) | 0.00 | -0.1% |
| N8 | Night shift | 408 | 3.86 (0.96 to 6.76) | 3.94 (1.03 to 6.86) | -0.08 | -2.2% |
| N8 | Orthopaedic involvement | 408 | 4.93 (2.42 to 7.44) | 5.28 (2.79 to 7.76) | -0.35 | -7.0% |

The descriptive percentage change is not a causal decomposition and no formal cross-outcome coefficient-difference test was performed. Abbreviations: CI, confidence interval; HC3, heteroskedasticity-consistent type 3.

**Supplementary Table S9. Subdomain and orthopaedic-subgroup analyses**

**Panel A. Exploratory subdomain analyses**

| **Outcome/domain** | **n** | **Night-shift β (HC3 95% CI)** | **P** | **Orthopaedic β (HC3 95% CI)** | **P** |
| --- | --- | --- | --- | --- | --- |
| General/common six-item score | 358 | 4.14 (-0.01 to 8.28) | 0.051 | 1.83 (-1.65 to 5.32) | 0.302 |
| Equal-weighted general/common score | 358 | 4.13 (-0.03 to 8.28) | 0.052 | 1.95 (-1.52 to 5.42) | 0.270 |
| Smoke-related three-item subdomain | 380 | 1.93 (-2.89 to 6.75) | 0.431 | 3.11 (-1.02 to 7.24) | 0.139 |
| C4.10 specialty-specific bone-cement/pulse-lavage score | 408 | -0.28 (-6.79 to 6.23) | 0.933 | 25.99 (19.54 to 32.44) | <0.001 |

The C4.10 specialty-specific score is expected to differ strongly by orthopaedic involvement and is not an independent validation analysis.

**Panel B. Model 1 analyses restricted to participants reporting orthopaedic work**

| **Outcome** | **n** | **Night-shift β (HC3 95% CI)** | **P** | **Interventional involvement β (HC3 95% CI)** | **P** |
| --- | --- | --- | --- | --- | --- |
| O10 original | 156 | 5.90 (0.42 to 11.37) | 0.035 | 3.14 (-2.62 to 8.90) | 0.283 |
| B9 excluding C4.10 | 155 | 6.29 (0.73 to 11.84) | 0.027 | 4.20 (-1.87 to 10.28) | 0.174 |
| G6 general/common | 135 | 7.31 (0.99 to 13.63) | 0.024 | 4.23 (-4.59 to 13.05) | 0.345 |

Orthopaedic involvement is invariant within this subgroup and is therefore not estimated. Abbreviations: CI, confidence interval; HC3, heteroskedasticity-consistent type 3.

**Supplementary Table S10. Progressive concern/attitude analyses and cognitive-variable diagnostics**

**Panel A. Same-sample progressive models (n=408)**

| **Focal factor** | **Stage** | **Adjustment** | **β (classical 95% CI)** | **P** | **β (HC3 95% CI)** | **P** |
| --- | --- | --- | --- | --- | --- | --- |
| Concern | A | Unadjusted | -0.57 (-1.78 to 0.63) | 0.350 | -0.57 (-1.72 to 0.57) | 0.326 |
| Concern | B | + age/sex | -0.40 (-1.61 to 0.81) | 0.517 | -0.40 (-1.56 to 0.76) | 0.500 |
| Concern | C | + recruitment group | 0.55 (-0.69 to 1.79) | 0.381 | 0.55 (-0.73 to 1.83) | 0.396 |
| Concern | D | + work-context variables | 0.79 (-0.43 to 2.02) | 0.202 | 0.79 (-0.48 to 2.06) | 0.220 |
| Concern | E | Full extended Model 2 | 1.41 (0.02 to 2.79) | 0.046 | 1.41 (-0.07 to 2.88) | 0.062 |
| Attitude | A | Unadjusted | 0.33 (-0.88 to 1.54) | 0.595 | 0.33 (-0.90 to 1.56) | 0.602 |
| Attitude | B | + age/sex | 0.46 (-0.75 to 1.68) | 0.453 | 0.46 (-0.80 to 1.73) | 0.471 |
| Attitude | C | + recruitment group | 0.60 (-0.58 to 1.77) | 0.321 | 0.60 (-0.72 to 1.91) | 0.374 |
| Attitude | D | + work-context variables | 0.66 (-0.49 to 1.81) | 0.261 | 0.66 (-0.62 to 1.94) | 0.313 |
| Attitude | E | Full extended Model 2 | 2.08 (0.54 to 3.61) | 0.008 | 2.08 (-0.22 to 4.38) | 0.077 |

For concern, the sign changed when recruitment-group fixed effects were introduced. Attitude was positive from the unadjusted model onward.

**Panel B. Pearson correlation matrix among standardised daily-life and cognitive-behavioural variables (n=408)**

| **Variable** | **Daily-life** | **Concern** | **Attitude** | **Norm** | **Peer** | **PBC** | **Intention** | **Protective** |
| --- | --- | --- | --- | --- | --- | --- | --- | --- |
| Daily-life | 1.00 | -0.10 | -0.10 | 0.06 | -0.04 | -0.09 | -0.13 | -0.17 |
| Concern | -0.10 | 1.00 | 0.31 | 0.32 | 0.44 | 0.45 | 0.42 | 0.39 |
| Attitude | -0.10 | 0.31 | 1.00 | 0.45 | 0.53 | 0.55 | 0.63 | 0.26 |
| Norm | 0.06 | 0.32 | 0.45 | 1.00 | 0.49 | 0.48 | 0.43 | 0.21 |
| Peer | -0.04 | 0.44 | 0.53 | 0.49 | 1.00 | 0.64 | 0.62 | 0.30 |
| PBC | -0.09 | 0.45 | 0.55 | 0.48 | 0.64 | 1.00 | 0.80 | 0.40 |
| Intention | -0.13 | 0.42 | 0.63 | 0.43 | 0.62 | 0.80 | 1.00 | 0.36 |
| Protective | -0.17 | 0.39 | 0.26 | 0.21 | 0.30 | 0.40 | 0.36 | 1.00 |

**Panel C. Full-model GVIF diagnostics**

| **Term** | **GVIF** | **df** | **Adjusted GVIF** |
| --- | --- | --- | --- |
| Age | 3.07 | 1 | 1.75 |
| Sex | 1.25 | 1 | 1.12 |
| Recruitment group | 2.78 | 8 | 1.07 |
| Operating-room work years | 2.61 | 1 | 1.62 |
| Night shift | 1.27 | 1 | 1.13 |
| Orthopaedics | 1.28 | 1 | 1.13 |
| Interventional | 1.17 | 1 | 1.08 |
| Daily-life | 1.18 | 1 | 1.09 |
| Concern | 1.58 | 1 | 1.26 |
| Attitude | 1.93 | 1 | 1.39 |
| Norm pressure | 1.59 | 1 | 1.26 |
| Peer influence | 2.11 | 1 | 1.45 |
| PBC | 3.44 | 1 | 1.86 |
| Intention | 3.52 | 1 | 1.88 |
| Protective behaviour | 1.35 | 1 | 1.16 |

Adjusted GVIF is GVIF^(1/(2×df)). No severe variance inflation was identified.

**Panel D. Natural-spline functional-form checks**

| **Focal factor** | **Stage** | **n** | **F** | **Added df** | **P** | **Holm-adjusted P** |
| --- | --- | --- | --- | --- | --- | --- |
| Concern | D | 408 | 0.42 | 2 | 0.658 | 1.000 |
| Concern | E | 408 | 0.71 | 2 | 0.494 | 1.000 |
| Attitude | D | 408 | 1.23 | 2 | 0.295 | 1.000 |
| Attitude | E | 408 | 1.35 | 2 | 0.259 | 1.000 |

No tested nonlinear term remained significant after Holm adjustment. Abbreviations: PBC, perceived behavioural control; GVIF, generalised variance inflation factor; df, degrees of freedom; SD, standard deviation.

**Supplementary Table S11. Night-shift frequency analyses**

**Panel A. Counts among 415 frontline participants**

| **Derivation** | **No night shift** | **<5/month** | **≥5/month** | **Missing** |
| --- | --- | --- | --- | --- |
| Main derivation | 97 | 210 | 101 | 7 |
| Conservative sensitivity | 93 | 210 | 101 | 11 |
| Directly recorded frequency only | 20 | 210 | 101 | 84 |

The structured questionnaire category was treated as ≥5/month according to the confirmed questionnaire classification; it was not interpreted as an exact count.

**Classification rules for the three frequency derivations:**

| **Derivation** | **No night shift** | **<5/month** | **≥5/month** | **Missing** |
| --- | --- | --- | --- | --- |
| Main | 20 explicit A9.1 'no night shift' + 73 explicit day-only schedules with A9.1 blank + 4 non-regular daytime records classified as no night shift from archived derived coding | 210 explicit A9.1 responses | 101 explicit A9.1 responses | 7 unresolved other-schedule records |
| Conservative sensitivity | 20 explicit A9.1 'no night shift' + 73 explicit day-only schedules | 210 explicit A9.1 responses | 101 explicit A9.1 responses | 7 unresolved + 4 non-regular daytime records |
| Directly recorded frequency only | 20 explicit A9.1 'no night shift' | 210 explicit A9.1 responses | 101 explicit A9.1 responses | 84 with A9.1 unavailable (73 day-only + 4 non-regular daytime + 7 unresolved) |

Derivation note. Four non-regular daytime records (fee-desk work, n=1; maternity leave, n=2; sick leave, n=1) were classified as no night shift in the main derivation based on archived derived coding. The conservative sensitivity derivation treated these four records as missing. Seven unresolved records remained missing in all derived classifications. The questionnaire category originally recorded as '5 or more' was analysed as ≥5/month according to the confirmed category meaning; it was not treated as an exact count.

**Panel B. Adjusted frequency coefficients**

| **Derivation** | **Contrast** | **Model n** | **β (classical 95% CI)** | **P** | **β (HC3 95% CI)** | **P** | **Global HC3 P** |
| --- | --- | --- | --- | --- | --- | --- | --- |
| Main derivation | <5/month vs no night | 408 | 3.15 (0.12 to 6.18) | 0.042 | 3.15 (0.11 to 6.19) | 0.042 | 0.016 |
| Main derivation | ≥5/month vs no night | 408 | 5.45 (1.83 to 9.08) | 0.003 | 5.45 (1.65 to 9.26) | 0.005 | 0.016 |
| Conservative sensitivity | <5/month vs no night | 404 | 3.61 (0.53 to 6.69) | 0.022 | 3.61 (0.55 to 6.66) | 0.021 | 0.008 |
| Conservative sensitivity | ≥5/month vs no night | 404 | 5.80 (2.15 to 9.44) | 0.002 | 5.80 (1.99 to 9.60) | 0.003 | 0.008 |
| Directly recorded frequency only | <5/month vs no night | 331 | 0.88 (-4.64 to 6.41) | 0.753 | 0.88 (-4.34 to 6.11) | 0.739 | 0.509 |
| Directly recorded frequency only | ≥5/month vs no night | 331 | 2.72 (-3.19 to 8.63) | 0.366 | 2.72 (-2.86 to 8.30) | 0.338 | 0.509 |

Models adjusted for age, sex, operating-room work years, orthopaedic involvement, interventional involvement, and recruitment-group fixed effects. No dose-response inference is made.

**Panel C. Model-adjusted occupational scenario-burden means**

| **Derivation** | **Category** | **Model n** | **Adjusted mean** | **HC3 95% CI** |
| --- | --- | --- | --- | --- |
| Main derivation | No night shift | 408 | 47.77 | 45.33 to 50.20 |
| Main derivation | <5/month | 408 | 50.92 | 49.25 to 52.58 |
| Main derivation | ≥5/month | 408 | 53.22 | 50.43 to 56.01 |
| Conservative sensitivity | No night shift | 404 | 47.42 | 44.97 to 49.87 |
| Conservative sensitivity | <5/month | 404 | 51.03 | 49.35 to 52.70 |
| Conservative sensitivity | ≥5/month | 404 | 53.22 | 50.43 to 56.00 |
| Directly recorded frequency only | No night shift | 331 | 50.33 | 45.50 to 55.17 |
| Directly recorded frequency only | <5/month | 331 | 51.22 | 49.55 to 52.89 |
| Directly recorded frequency only | ≥5/month | 331 | 53.05 | 50.14 to 55.97 |

Abbreviations: CI, confidence interval; HC3, heteroskedasticity-consistent type 3.

**Supplementary Table S12. Exploratory binary outcome models and absolute effect measures**

**Panel A. Binary sensitivity models**

| **Outcome/model** | **Associated factor** | **n** | **Events** | **OR (95% CI)** | **P** | **CI method** |
| --- | --- | --- | --- | --- | --- | --- |
| Upper tertile (>54.0541), Model 1 | Any night shift | 408 | 135 | 2.16 (1.16 to 3.99) | 0.015 | Wald |
| Upper tertile (>54.0541), Model 1 | Orthopaedic involvement | 408 | 135 | 1.54 (0.94 to 2.53) | 0.088 | Wald |
| Upper tertile (>54.0541), Model 2 | Any night shift | 408 | 135 | 2.06 (1.09 to 3.89) | 0.026 | Wald |
| Upper tertile (>54.0541), Model 2 | Orthopaedic involvement | 408 | 135 | 1.58 (0.95 to 2.61) | 0.076 | Wald |
| Frontline upper quartile (>58.8235), Model 1 | Any night shift | 408 | 99 | 3.84 (1.67 to 8.83) | 0.002 | Wald |
| Frontline upper quartile (>58.8235), Model 1 | Orthopaedic involvement | 408 | 99 | 2.47 (1.42 to 4.31) | 0.001 | Wald |
| Frontline upper quartile (>58.8235), Model 2 | Any night shift | 408 | 99 | 3.46 (1.48 to 8.13) | 0.004 | Wald |
| Frontline upper quartile (>58.8235), Model 2 | Orthopaedic involvement | 408 | 99 | 2.55 (1.45 to 4.50) | 0.001 | Wald |

Ties at each cut-off remained in the lower group. Neither cut-off represents a clinical, exposure, or safety threshold.

The upper-quartile sensitivity used the frontline-specific 75th percentile (58.8235), with ties at the cut-off retained in the lower group.

**Panel B. Model-standardised probabilities and contrasts for the upper-tertile outcome**

| **Model** | **Factor** | **n** | **Reference=0 probability** | **Factor=1 probability** | **Absolute difference** | **Probability ratio** |
| --- | --- | --- | --- | --- | --- | --- |
| Model 1 | Night shift | 408 | 22.0% (13.3–30.8) | 36.3% (31.1–41.5) | 14.3 pp (3.8 to 24.7) | 1.65 (1.07 to 2.54) |
| Model 1 | Orthopaedic involvement | 408 | 29.6% (24.0–35.3) | 38.3% (30.7–45.9) | 8.6 pp (-1.4 to 18.6) | 1.29 (0.97 to 1.73) |
| Model 2 | Night shift | 408 | 22.8% (13.7–31.8) | 36.1% (30.9–41.2) | 13.3 pp (2.4 to 24.1) | 1.58 (1.02 to 2.45) |
| Model 2 | Orthopaedic involvement | 408 | 29.5% (23.9–35.1) | 38.5% (30.9–46.1) | 9.0 pp (-1.0 to 19.0) | 1.30 (0.98 to 1.74) |

OR, odds ratio; CI, confidence interval; pp, percentage points. These are model-standardised associations under the observed covariate distribution, not causal intervention effects.

**Supplementary Table S13. Binary-model complexity and internal stability diagnostics**

**Panel A. Binary-model complexity and internal bootstrap diagnostics**

| **Model** | **n** | **Events** | **Non-intercept parameters** | **Events/parameter** | **AIC** | **Apparent AUC** | **Corrected AUC** | **Apparent Brier** | **Corrected Brier** | **Corrected calibration slope** |
| --- | --- | --- | --- | --- | --- | --- | --- | --- | --- | --- |
| Model 1 | 408 | 135 | 14 | 9.64 | 499.81 | 0.693 | 0.647 | 0.196 | 0.211 | 0.750 |
| Model 2 | 408 | 135 | 22 | 6.14 | 510.44 | 0.711 | 0.641 | 0.193 | 0.217 | 0.643 |

Bootstrap correction used 300 recruitment-group-stratified resamples. Corrected values are internal diagnostics, not external validation.

**Panel B. Ridge-logistic coefficient shrinkage sensitivity**

| **Model** | **Night-shift OR** | **Orthopaedic OR** | **λ1se** | **Tuning** |
| --- | --- | --- | --- | --- |
| Model 1 | 1.45 | 1.25 | 0.237 | 5-fold CV; λ1se |
| Model 2 | 1.23 | 1.14 | 0.547 | 5-fold CV; λ1se |

Ridge models were used only to assess coefficient shrinkage; conventional P values and confidence intervals were not assigned to penalised estimates.

**Panel C. Sparse-data and boundary-fit assessment**

| **Issue** | **Location/model** | **Finding** |
| --- | --- | --- |
| Sparse complete-case upper-tertile cells | Group G | 4 events |
| Bootstrap pure-outcome recruitment-group draws | Model 1 | 0 of 300 resamples |
| Bootstrap pure-outcome recruitment-group draws | Model 2 | 0 of 300 resamples |
| Alternative binary models with separation | Corrected 9-group binary designs | 0 of 40 designs flagged by independent LP separation check |

Independent linear-programming checks identified no separation in the corrected nine-group binary designs; bootstrap boundary diagnostics likewise found no pure-outcome recruitment-group draws in the 300 resamples per model. Software convergence was not used as the sole criterion for finite maximum-likelihood estimates.

**Panel D. Binary-model fit comparison**

| **Model** | **n** | **AIC** | **Null deviance** | **Residual deviance** |
| --- | --- | --- | --- | --- |
| Model 1: occupational structure | 408 | 499.81 | 518.00 | 469.81 |
| Model 2: extended behavioural model | 408 | 510.44 | 518.00 | 464.44 |

Likelihood-ratio test comparing Model 1 with Model 2: deviance difference 5.36, df=8, P=0.718. This fit comparison is supplementary to the principal continuous analysis; the binary models are exploratory sensitivity analyses. Abbreviations: AIC, Akaike information criterion; AUC, area under the receiver operating characteristic curve; OR, odds ratio; CV, cross-validation; df, degrees of freedom; λ1se, penalisation parameter selected by the one-standard-error rule.

**Supplementary Table S14. Recruitment-group robustness analyses**

**Panel A. Random-intercept diagnostics**

| **Model** | **n** | **Groups** | **Group variance** | **Residual/latent variance** | **ICC** | **Converged** | **Singular** |
| --- | --- | --- | --- | --- | --- | --- | --- |
| Continuous Model 1 | 408 | 9 | 15.398 | 130.814 | 0.105 | TRUE | FALSE |
| Continuous Model 2 | 408 | 9 | 13.224 | 126.999 | 0.094 | TRUE | FALSE |
| Binary Model 1 | 408 | 9 | 0.292 | 3.290 | 0.081 | TRUE | FALSE |
| Binary Model 2 | 408 | 9 | 0.248 | 3.290 | 0.070 | TRUE | FALSE |

Intraclass correlation coefficient (ICC) denotes between-recruitment-group heterogeneity, not hospital-level ICC, because Group C/J is one heterogeneous visiting-nurse cohort comprising nurses from multiple home institutions.

**Panel B. Key random-intercept sensitivity estimates**

| **Model** | **Factor** | **β or OR (Wald 95% CI)** | **P** |
| --- | --- | --- | --- |
| Continuous Model 1 | Night shift | 3.90 (1.05 to 6.74) | 0.007 |
| Continuous Model 1 | Orthopaedic involvement | 4.92 (2.39 to 7.45) | <0.001 |
| Continuous Model 2 | Night shift | 3.11 (0.23 to 5.99) | 0.034 |
| Continuous Model 2 | Orthopaedic involvement | 4.84 (2.33 to 7.35) | <0.001 |
| Binary Model 1 | Night shift | 2.16 (1.18 to 3.96) | 0.013 |
| Binary Model 1 | Orthopaedic involvement | 1.50 (0.93 to 2.42) | 0.098 |
| Binary Model 2 | Night shift | 2.03 (1.09 to 3.79) | 0.026 |
| Binary Model 2 | Orthopaedic involvement | 1.51 (0.93 to 2.46) | 0.094 |

**Panel C. Leave-one-recruitment-group-out ranges**

| **Outcome type** | **Factor** | **Models** | **Range of point estimates** |
| --- | --- | --- | --- |
| Continuous | Night shift | 9 | 2.41 to 5.25 |
| Continuous | Orthopaedic involvement | 9 | 4.36 to 5.95 |
| Binary | Night shift | 9 | 1.82 to 2.63 |
| Binary | Orthopaedic involvement | 9 | 1.37 to 1.90 |

All point estimates remained positive, but not all confidence intervals excluded the null. The tertile threshold was not recalculated after group exclusion. Abbreviations: OR, odds ratio; CI, confidence interval.

**Supplementary Table S15. Survey-mode characteristics and online-only sensitivity analyses**

**Panel A. Descriptive characteristics by administration mode**

| **Mode** | **Frontline n** | **O10 mean (SD)** | **O10 median (Q1–Q3)** | **Age mean (SD)** | **Female n (%)** | Mean operating-room work-years category |
| --- | --- | --- | --- | --- | --- | --- |
| Paper | 69 | 55.9 (11.5) | 54.1 (48.5–62.2) | 32.0 (5.6) | 57 (82.6) | 2.67 |
| Online | 346 | 49.7 (12.3) | 51.4 (40.5–56.8) | 31.3 (6.4) | 292 (84.4) | 2.41 |

These comparisons are descriptive only. Paper administration was completely nested within recruitment Groups D and F; observed differences cannot be attributed independently to survey mode.

**Panel B. Selected participant characteristics by administration mode**

| **Mode** | **Scrub/instrument** | **Circulating** | **Anaesthesia monitoring** | **Orthopaedics** | **Interventional** | **General surgery** |
| --- | --- | --- | --- | --- | --- | --- |
| Paper | 51 (73.9) | 58 (84.1) | 0 (0.0) | 25 (36.2) | 6 (8.7) | 39 (56.5) |
| Online | 233 (67.3) | 228 (65.9) | 10 (2.9) | 136 (39.3) | 40 (11.6) | 194 (56.1) |

Values are n (%). These are descriptive comparisons; roles/specialties were multiple-response items.

**Panel C. Non-scorable occupational-item responses by mode**

| **Item** | **Scenario** | **Paper n (%)** | **Online n (%)** |
| --- | --- | --- | --- |
| C4.1 | Medical plastic-item handling | 0 (0.0) | 0 (0.0) |
| C4.2 | Disposable gown duration | 0 (0.0) | 0 (0.0) |
| C4.3 | Disposable cap use | 0 (0.0) | 0 (0.0) |
| C4.4 | Sterile-package opening | 10 (14.5) | 40 (11.6) |
| C4.5 | Surgical draping | 0 (0.0) | 0 (0.0) |
| C4.6 | PVC glove use | 0 (0.0) | 0 (0.0) |
| C4.7 | Surgical smoke occurrence | 0 (0.0) | 0 (0.0) |
| C4.8 | Immediate smoke evacuation | 0 (0.0) | 0 (0.0) |
| C4.9 | Laparoscopic smoke management | 10 (14.5) | 19 (5.5) |
| C4.10 | Bone cement/pulse-lavage work | 0 (0.0) | 0 (0.0) |

Non-scorable does not uniformly mean missing or non-applicable; see Supplementary Table S5.

**Panel D. Online-only sensitivity analyses**

| **Analysis** | **Factor** | **n** | **Effect** | **Estimate (95% CI)** | **P** |
| --- | --- | --- | --- | --- | --- |
| O10 continuous Model 1 | Night shift | 340 | β | 3.67 (0.34 to 7.01) | 0.031 |
| O10 continuous Model 1 | Orthopaedic involvement | 340 | β | 4.88 (2.16 to 7.60) | <0.001 |
| B9 continuous Model 1 | Night shift | 335 | β | 4.43 (0.94 to 7.92) | 0.013 |
| B9 continuous Model 1 | Orthopaedic involvement | 335 | β | 2.04 (-0.78 to 4.85) | 0.155 |
| O10 upper-tertile Model 1 | Night shift | 340 | OR | 2.12 (1.03 to 4.38) | 0.042 |
| O10 upper-tertile Model 1 | Orthopaedic involvement | 340 | OR | 1.64 (0.94 to 2.85) | 0.081 |

Excluding paper-only Groups D and F also changes recruitment-group composition; these analyses therefore do not isolate a pure administration-mode effect.

Interval note. Continuous-effect 95% confidence intervals in Panel D use HC3 heteroskedasticity-consistent inference. Upper-tertile logistic-model 95% confidence intervals in Panel D are Wald intervals. Abbreviations: O10, original 10-item occupational index; B9, O10 excluding C4.10; Q1–Q3, first to third quartiles; OR, odds ratio; CI, confidence interval; HC3, heteroskedasticity-consistent type 3.

**Supplementary Table S16. Descriptive content analysis of open-ended comments**

**Panel A. Open-ended response status**

| **Open-ended response status** | **n** | **% of eligible respondents** |
| --- | --- | --- |
| Blank response | 274 | 61.2 |
| Non-substantive response | 148 | 33.0 |
| Substantive suggestion included in content analysis | 26 | 5.8 |
| Total | 448 | 100.0 |

Only the 26 substantive comments were coded thematically. The open-ended component is a limited descriptive supplement from a small self-selected subset.

**Panel B. Themes identified from 26 substantive comments**

| **Theme** | **Operational definition** | **n** | **%** | **Representative de-identified quote** | **Descriptive relevance** |
| --- | --- | --- | --- | --- | --- |
| Education and awareness | Training, health education, knowledge dissemination, awareness-raising or prevention education | 11 | 42.3 | “Strengthen education and publicity about the hazards of microplastics.” | Education/training area for organisational assessment |
| Material substitution and plastic reduction | Reduce disposable plastic use, replace materials, or reduce unnecessary plastic consumables | 8 | 30.8 | “Reduce the use of disposable items.” | Procurement/material-use area for assessment |
| Occupational health monitoring and protection | Personal/occupational protection, safer protective products, or staff health-protection measures | 5 | 19.2 | “Improve the quality of disposable protective equipment.” | Occupational-health/protection area for assessment |
| Institutional policy and procurement management | Management-level action, policy, procurement standards, leadership support, or standardised consumable use | 5 | 19.2 | “This should mainly be solved at the management level.” | Policy/procurement area for assessment |
| Ventilation, smoke evacuation and environmental control | Surgical smoke, exhaust systems, ventilation, air quality, or environmental controls | 4 | 15.4 | “Use suction to remove electrocautery smoke.” | Environmental-control area for assessment |
| Food, water and rest-area infrastructure | Drinking water, bottled water, cups, meal containers, canteen utensils, or rest-area infrastructure | 4 | 15.4 | “Provide purified direct drinking water.” | Workplace-infrastructure area for assessment |
| Waste sorting and recycling | Recycling, reuse, circular material use, or waste reduction | 2 | 7.7 | “Use materials that are harmless to the body and can be effectively recycled.” | Environmental-management area for assessment |
| Other | Meaningful comments not fitting the above themes | 2 | 7.7 | “Improve facilities.” | Broader facility-level assessment |
| Staffing, workload and night-shift support | Staffing, workload, rest, night-shift support, or scheduling | 0 | 0.0 | Not reported | No substantive comment directly addressed this theme |

A single comment could be coded to more than one theme; percentages therefore do not sum to 100%. Quotes were translated from Chinese and de-identified. Theme frequencies do not independently confirm structured survey findings.

**Supplementary Table S17. Regression diagnostic checks for the principal continuous and exploratory upper-tertile models**

**Panel A. Principal continuous O10 models**

| **Diagnostic** | **Model 1** | **Model 2** | **Interpretation** |
| --- | --- | --- | --- |
| Model sample | n=408 | n=408 | Principal complete-case continuous models |
| Breusch–Pagan heteroskedasticity test | χ²=28.32; P=0.013 | χ²=37.68; P=0.020 | Evidence of heteroskedasticity supported HC3 inference alongside conventional OLS estimates. |
| Cook's D >4/n | 21 observations | 21 observations | Used as the case-deletion influence sensitivity set. |
| Leverage >2p/n | 19 observations | 13 observations | Screening diagnostic; p includes the intercept. |
| Fitted O10 values outside 0–100 | 0 | 0 | No fitted value fell outside the index range. |
| Cook-deletion night-shift estimate | β=3.28 (0.69 to 5.88); n=387 | β=2.88 (0.24 to 5.52); n=387 | HC3 95% CI after excluding Cook-flagged observations. |
| Cook-deletion orthopaedic estimate | β=4.31 (2.03 to 6.60); n=387 | β=4.31 (2.06 to 6.57); n=387 | HC3 95% CI after excluding Cook-flagged observations. |
| Functional-form checks | Age spline P=.573; work-years alternative P=.058 (Holm P=.116) | No tested nonlinear term significant after Holm adjustment; smallest raw P=.077 (Holm P=.768) | Natural-spline checks were used for continuous covariates; work years was also compared with a categorical specification. |

Residual diagnostics. Standard residual-versus-fitted, normal quantile–quantile (Q–Q), scale-location, and residual-versus-leverage plots were reviewed for both continuous O10 models. The Breusch–Pagan tests detected heteroskedasticity; inference therefore emphasised HC3 confidence intervals and P values. Influence screening also examined maximum absolute DFBETA >2/√n; the compact case-deletion sensitivity shown above used Cook's D >4/n.

**Panel B. Exploratory upper-tertile logistic models**

| **Diagnostic** | **Model 1** | **Model 2** | **Interpretation** |
| --- | --- | --- | --- |
| Model sample / events | n=408 / 135 events | n=408 / 135 events | Upper-tertile sensitivity outcome (>54.0541). |
| Cook's D >4/n | 17 observations | 23 observations | Used as the case-deletion influence sensitivity set. |
| Leverage >2p/n | 26 observations | 15 observations | Screening diagnostic; p includes the intercept. |
| Extreme fitted probability (<10⁻⁶ or >1−10⁻⁶) | 0 | 0 | No extreme fitted probability in the original O10 upper-tertile models. |
| Cook-deletion night-shift estimate | OR=3.04 (1.47 to 6.28); n=391 | OR=3.22 (1.47 to 7.06); n=385 | Wald 95% CI after excluding Cook-flagged observations. |
| Cook-deletion orthopaedic estimate | OR=1.71 (0.99 to 2.95); n=391 | OR=1.71 (0.97 to 3.00); n=385 | Wald 95% CI after excluding Cook-flagged observations. |
| Functional-form checks | Age P=.362; work-years alternative P=.301; Holm P≥.602 | Smallest raw P=.033 for norm pressure, Holm P=.328; all tested terms nonsignificant after Holm adjustment | Likelihood-ratio comparisons for spline/categorical alternatives. |

Diagnostic scope. Panel B complements the bootstrap, calibration, ridge, sparse-cell and boundary-fit diagnostics already reported in Supplementary Table S13. Functional-form checks did not identify a Holm-adjusted nonlinear term. These diagnostics support sensitivity assessment of model specification and influence; they are not external validation. Abbreviations: O10, original 10-item occupational index; OR, odds ratio; CI, confidence interval; HC3, heteroskedasticity-consistent type 3; OLS, ordinary least squares; DFBETA, change in a regression coefficient after deleting one observation.

**Supplementary Figure S1. Distributions of the continuous occupational and daily-life scenario-burden indices among frontline operating-room nursing staff.**

Figure note. The continuous occupational scenario-burden score is the principal analytical representation. In Panel A, the dashed and dotted vertical reference lines mark the sample-derived upper-tertile (>54.0541) and frontline-specific upper-quartile (>58.8235) cut-offs, respectively; ties at each cut-off remain in the lower group. The shaded region denotes the exploratory upper-tertile group (n=138). Both cut-offs are used as within-sample sensitivity definitions of the continuous principal outcome. Panel B shows the daily-life scenario-burden distribution without a threshold.

**Questionnaire files.** The participant questionnaires are supplied separately as Supplementary File 1 (Chinese questionnaire, anonymised) and Supplementary File 2 (English reporting translation). The English version was prepared for reporting purposes and was not used for data collection.
