# Supplementary material for: Self‐Reported Plastic‐Related Occupational Scenarios and Perceived Support Priorities Among Operating‐Room Nursing Staff: A Multicentre Cross‐Sectional Survey
Source: J Nurs Manag. 2026 Oct 17;2026:7475740. doi: 10.1155/jonm/7475740 (PMC13675910; doi:10.1155/jonm/7475740)
Supplement: Supplementary file 1 [file JONM-2026-7475740-s001.zip › Supplementary_File_1_Chinese_questionnaire.pdf]

手术室护理人员微塑料暴露风险多中心调查

中文问卷（研究中心匿名化版本）

匿名化说明

本补充文件为中文问卷的投稿匿名化版本。真实研究中心名称已替换为 Site A、Site B、Site C 或“其他参与中心”。
伦理审查机构名称已处理为“牵头研究中心伦理委员会”，以避免在问卷补充材料中暴露研究中心身份。
问卷中的员工/胸牌号字段仅用于说明原始问卷设计；正式分析和共享数据集中已删除直接识别信息。

（多中心研究牵头单位已匿名化）

尊敬的护理同仁：

您好！我们是“医疗机构手术室护理人员微塑料暴露风险多中心调查”课题组，诚挚邀请您参与本次研究。 手机扫描即可填写

微塑料是广泛存在于环境中的微小塑料颗粒。手术室环境中，护理人员可能通过多种途径接触到微塑料。本研究旨在了解手术室护理人员微塑料暴露的潜在风险、认知水平及防护行为，成果将为制定针对性的职业防护指南、提升医护职业健康水平提供至关重要的科学依据。

本研究已通过牵头研究中心伦理委员会审查[2025 年审（066）号]。本问卷采用匿名方式，所有信息将被严格保密，数据仅用于整体分析。完成问卷大约需要 15-20 分钟。您的参与完全基于自愿，但您的每一个回答都至关重要。

我已阅读并理解上述信息，确认我的所有疑问已得到解答。我自愿同意参与本研究。 （多选）
请勾选以下选项表明您的意愿：

我确认已阅读并理解上述内容，我自愿参与本次问卷调查，我知晓可在提交后 7 日内通过邮件撤回数据（需提供撤回方式）

第一部分：基础信息

本部分信息对于本研究至关重要，所有内容将被严格保密，仅用于整体数据分析。请您根据实际情况填写。

A1. 您目前所在的工作单位 （单选）

□ Site A
□ Site B
□ Site C

□ 其他参与中心（请填写/选择匿名编号或机构类别）____________________________

A2. 您的员工/胸牌号（仅用于数据核对；数据分析前删除）

____

____

____

A3. 性别 （单选）

□ 男
□ 女

A4. 年龄

____

A5. 身高与体重 (用于健康指标分析)

数值

身高 cm

体重 kg

A6. 您在手术室的工作年限 （单选）

□ <1 年
□ 1-3 年

□ 4-6 年
□ 7-10 年
□ ＞10 年

A7. 您在手术室内的主要岗位角色 (可多选) （多选）

□ 巡回护士
□ 器械护士/洗手护士
□ 麻醉监测护士

□ 其他非手术岗位(管理人员、收费审核等，请注明) ______________________________

A8. 您主要参与的手术类型 （多选选 1-12 项）

□ 普外
□ 骨科
□ 体外（心脏外科）
□ 神经
□ 介入手术

□ 妇科/泌尿
□ 耳鼻喉
□ 甲乳
□ 胸外

□ 整形
□ 眼科

□ 其他（非手术岗位，请填写具体工作内容如：收费审核/无菌室管理/质控/排程）
____

A9. 您目前主要的工作班制属于以下哪一种 （单选）

仅日间白班 (通常为行政班，无夜间值班)

轮班制 (包含夜班、接班、二线等)

□ 其他（请注明） ________________________________

A9.1. 您平均每月的夜班频次约为 （单选）

□ 少于 5 个

5 个以上

□ 不涉及夜班

A10. 您的最高学历 （单选）

□ 中专
□ 大专
□ 本科
□ 研究生及以上

A11. 您的职称 （单选）

□ 护士
□ 护师
□ 主管护师
□ 副主任护师及以上

第二部分：微塑料认知和关注度

*本部分旨在了解您对"微塑料"相关知识的熟悉程度及个人关注兴趣。所有问题均无对错之分，调查结果将严*格保密，请根据您的真实情况填写。

填写说明： 请使用1-10 分 评估您对下列内容的了解程度与关注程度。

1 分=不了解 10 分=非常了解

1 分=不关注 10 分=非常关注

B1. 请评估您对以下基础概念的认知与关注程度：

B1.1 参加本次微塑料问卷前，您对于“微塑料”的了解和关注程度？

指环境中粒径小于 5 毫米的塑料颗粒、纤维或碎片。例如：磨砂膏中的颗粒、瓶装水可能含有的微小塑料。

认知程度 1 2 3 4 5 6 7 8 9 10

关注程度 1 2 3 4 5 6 7 8 9 10

B1.2 参加本次微塑料问卷前，您对于 PCCPs（个人护理与化妆品）中的微塑料的认知和关注程

度？

PCCPs 是个人护理和化妆品产品的缩写。此类产品可能有意添加塑料微珠（如磨砂洗面奶、牙膏、沐浴露）中的塑料微珠或聚合物成分。其主要功能包括磨砂、增稠、增加顺滑感等，成分表中常标注为聚乙烯（PE）、聚

丙烯（PP）或聚季铵盐等。

认知程度 1 2 3 4 5 6 7 8 9 10

关注程度 1 2 3 4 5 6 7 8 9 10

B1.3 参加本次微塑料问卷前，您对于医疗环境中的微塑料释放的认知和关注程度？

指在医疗操作过程中，塑料制品释放微塑料的可能性。例如频繁摩擦或开启塑料包装的注射器/器械、防护服/鞋套的磨损，手术烟雾气溶胶、空气中飘浮的合成纤维等。

认知程度 1 2 3 4 5 6 7 8 9 10

关注程度 1 2 3 4 5 6 7 8 9 10

B2. 请评估您对以下微塑料健康风险的认知与关注程度：

B2.1 参加本次微塑料问卷前，您对微塑料引发的炎症反应

研究表明，微塑料进入人体后可能激活免疫细胞异常，从而引发局部或全身性的慢性炎症。

认知程度 1 2 3 4 5 6 7 8 9 10

关注程度 1 2 3 4 5 6 7 8 9 10

B2.2 参加本次微塑料问卷前，您对微塑料与心血管疾病的关联

最新临床研究发现，颈动脉斑块中检测到微塑料的患者，其后续发生心肌梗死、卒中或死亡的风险比（OR 值）

□ 是不含微塑料患者的 4.5 倍。

认知程度 1 2 3 4 5 6 7 8 9 10

关注程度 1 2 3 4 5 6 7 8 9 10

B3. 请评估您对以下微塑料防护措施的认知与关注程度：

B3.1 参加本次微塑料问卷前，您了解的 煮沸自来水的去除微塑料效果
研究显示，将自来水煮沸后过滤，可去除水中约 90% 的纳/微米塑料。这是一种简单有效的日常防护方法。

认知程度 1 2 3 4 5 6 7 8 9 10

关注程度 1 2 3 4 5 6 7 8 9 10

B3.2 参加本次微塑料问卷前，您对天然材质容器的替代价值
使用玻璃、不锈钢、陶瓷等天然材质的容器，替代塑料杯、塑料饭盒，可以从源头上显著减少微塑料在饮食过程中的溶出和摄入。

认知程度 1 2 3 4 5 6 7 8 9 10

关注程度 1 2 3 4 5 6 7 8 9 10

第三部分：暴露途径评估

*本部分将详细调查您在日常生活和手术室职业环境中可能接触微塑料的途径与频率。您的详细回答将帮助我们*精准识别关键暴露环节。

日常生活暴露 C1 饮水习惯调查 (请回顾您过去一个月的情况)

C 1.1 您所在手术室的饮用水主要来源是？ （单选）

□ 桶装水+饮水机
□ 管道直饮水（连接医院净水系统）
□ 仅提供瓶装水

□ 其他（请说明）____________________________

C 1.2 工作中（在医院期间）您最常使用的饮水容器主要材质是？ （单选）
(请思考您最常用的杯子或水壶)

□ 塑料杯/壶
□ 不锈钢杯/壶

□ 玻璃杯/陶瓷杯
□ 一次性纸杯
□ 随场景变化，无固定容器

C1.3 工作外（院外）您最常使用的饮水容器主要材质是？（单选）
(请思考您最常用的杯子或水壶)

□ 塑料杯/壶
□ 不锈钢杯/壶

□ 玻璃杯/陶瓷杯
□ 一次性纸杯
□ 随场景变化，无固定容器

C1.4 过去一个月工作中（在医院期间）您饮用塑料瓶装水、瓶装饮料以及外卖饮品（如奶茶、
咖啡）的频率？（单选）

□ 每天多次
□ 每天 1 次

□ 每周 2-3 次
□ 很少喝（每周<1 次）

□ 从不喝

C1.5 过去一个月工作外（院外）您饮用塑料瓶装水、瓶装饮料以及外卖饮品（如奶茶、咖啡）
的频率？（单选）

□ 每天多次
□ 每天 1 次

□ 每周 2-3 次
□ 很少喝（每周<1 次）

□ 从不喝

C1.6 过去一个月工作中（在医院期间），您使用一次性纸杯饮水的频繁程度？（单选）
□ 总是使用（几乎每次都是）
□ 经常使用（大部分时候是）
□ 有时使用（大约一半的情况）
□ 很少使用（偶尔）
□ 从不使用（我只使用自己的水杯）

C1.7 过去一个月工作之外（院外），您使用一次性纸杯饮水的频繁程度？（单选）
□ 总是使用（几乎每次都是）

□ 经常使用（大部分时候是）
□ 有时使用（大约一半的情况）
□ 很少使用（偶尔）
□ 从不使用（我只使用自己的水杯）

日常生活暴露 C2. 饮食习惯详细调查 (请回顾您过去一个月的情况)

C2.1 过去一个月工作中 (工作餐、值班餐、点外卖等)您使用塑料餐盒、塑料袋或保鲜膜直接盛装热食或热菜的频率是？ （单选）
(包括用微波炉加热塑料餐盒，或直接盛装刚出锅的热菜、热汤)

□ 很高 (>80%的时间)

□ 较高 (50%-80%的时间)
□ 一般 (30%-50%的时间)
□ 较低 (<30%的时间)
□ 从不

C2.2 过去一个月工作外 （包括居家、外出就餐、点外卖等），您使用使用塑料餐盒、塑料袋或保鲜膜直接盛装热食或热菜的频率是？ （单选）
(包括用微波炉加热塑料餐盒，或直接盛装刚出锅的热菜、热汤)
□ 很高 (>80%的时间)

□ 较高 (50%-80%的时间)
□ 一般 (30%-50%的时间)
□ 较低 (<30%的时间)

□ 从不

日常生活暴露 C3. 个人护理及用品使用习惯

C3.1 牙膏：您是否使用含有塑料微珠（成分表常标注为聚乙烯 PE、聚丙烯 PP） 的牙膏？ （单选）
□ 是，我常用且知晓其含微珠

□ 我常用，但不清楚是否含微珠
□ 我特意选择不含微珠的牙膏
□ 不固定，未关注过此问题

C3.2 磨砂洗面奶/去角质膏：您使用含塑料磨砂颗粒的洁面产品的频率是？ （单选）
产品名称中可能包含：深层洁净磨砂膏、去角质洁面乳、柔珠洁面乳，成分表常标注为：聚乙烯 PE、聚丙烯

PP、聚对苯二甲酸乙二醇酯（PET）

□ 每天使用
□ 每周使用 3-5 次

□ 每周使用 1-2 次
□ 偶尔使用（每月几次）

□ 从不使用

职业环境暴露（手术室） C4. 手术室医疗特有暴露评估

C4.1 最近一月中，平均每天需要进行塑料物品操作和处理频率是？ 包括但不限于：配置药
液、挤压输液袋、连接/拆卸注射器或延长管、三通阀等操作。 （单选）
□ 很少接触
□ 一般（<10 次/天）

□ 较高（<20 次/天）
高频（20-30 次/天）
非常高频（>30 次/天）

C4.2 最近一月中，平均每个白班中，您穿戴一次性手术衣的累计总时长大约是？ （单选）
E. 几乎不穿
□ 少于 2 小时

2 至 4 小时（含 2 小时）
4 至 6 小时（含 4 小时）

□ 超过 6 小时

C4.3 您工作中戴一次性手术帽的频率是？ （单选）
□ 总是
□ 经常

□ 有时
□ 偶尔
□ 几乎不

C4.4 最近一月中，平均每个白班中，您平均需要打开多少个一次性无菌物品（敷料包、器械
包、各类耗材）的塑料外包装？ （单选）
□ 很少（1-30 个）
较多（31-60 个）

很多（61-90 个）
非常多（90 个以上）
□ 不固定，差异极大/难以估计

C4.5 最近一月中，平均每个白班中，您平均需要进行多少台手术的铺巾？（单选）
较少（1-2 台）
中等（3-5 台）

较多（5-9 台）
极多（10 台以上）
□ 不涉及

C4.6 最近一月中，平均每个白班中，您平均使用一次性薄膜手套或 PVC 手套的数量是？（单
选）
□ 5 双及以上
□ 3-4 双

□ 1-2 双
□ 0 双（使用其他防护或直接操作）

C4.7 最近一月中，在您参与的手术中，因使用能量设备导致塑料器械或敷料灼烧产生可见或可
闻烟雾的情况，发生的频率是？（单选）
主要指在使用电刀、超声刀等能量设备进行手术时产生的烟雾，高温使人体组织和与之接触的塑料器械（如套管、超声刀头的塑料片）、敷料、包装等物质发生热解或汽化，其中可能包含汽化的塑料成分。
□ 经常发生（≥50%的手术中）
□ 有时发生（约 10%-50%的手术中）
□ 偶尔发生（＜10%的手术中）
□ 几乎从未发生
□ 不涉及此类手术

C4.8 在开放手术中，使用电刀、超声刀等设备产生明显烟雾时，您所在团队能立即使用吸引器
或高效烟雾吸引器的频繁程度是？（单选）
□ 总是能立即使用
□ 经常能立即使用
大约一半的情况能
□ 很少能立即使用
□ 几乎从不使用 / 设备常备但反应不及

C4.9 在腔镜手术中，使用电刀、超声刀等设备产生明显烟雾时，您所在团队通常会如何处置？
（单选）
□ 【常规主动防护】 使用带负压吸引或自动排烟器通过穿刺器阀门连接吸引器

□【经常主动防护】经常使用带负压吸引的排气方式，但有时会疏忽。
□【视情况混合使用】视烟雾大小和设备情况而定，有时吸引，有时直接排放到空气中。
□【常规被动释放】通常直接打开阀门排放到手术间空气中。
□【不涉及/无固定模式】不进行此类手术 / 方式不固定，完全取决于主刀医生习惯。

C4.10 最近一月中，在您手术配合过程中，涉及骨水泥、脉冲等操作频率？ （单选）

□ 每天≥1 次
□ 每周 2-4 次
□ 每周 1 次

□ 偶尔（每月几次）
□ 几乎不涉及

医院支持与后勤保障（手术室）

C5.1 您所在的手术室/医院，为值班人员提供餐食的主要模式是？ （单选）

□ 医院食堂统一配餐（使用医院统一的餐盒打包送至手术室）
□ 合作外卖定制（医院与特定餐饮公司合作，由其统一配送）
□ 无统一安排，完全自行解决（需自己点外卖或自带）

□ 混合模式（请简要说明） ______________________________

C5.2 值班室提供的被褥、枕头，您感觉其填充物主要是？ （单选）

□ 化纤棉（如七孔棉、PP 棉）

□ 天然材料(如棉花、羽绒）
□ 不清楚
□ 不提供

C5.3 您所在的手术室，为医护人员常规提供的帽子主要属于以下哪种类型？ （单选）

□ 一次性无纺布帽子（最常见，颜色多样，质地较挺括）
□ 一次性医用纸帽（质感像纸张，透气，易褶皱）

□ 可重复使用的纯棉布帽子（棉质，通常由医院统一清洗消毒）
□ 可重复使用的化纤布帽子（如聚酯纤维等合成面料）
混合提供或类型不固定

第四部分：态度与行为意向 导语： 本部分旨在了解您对于“减少微塑料暴露”这一行为的看

法、感受和计划。请根据您的真实想法，判断您对下列陈述的同意程度。答案无对错之分，调查结果将严格保密。 填写说明： 请使用 1-10 分 表明您的态度。 1 = 强烈不同意 2-3= 不同意

4-5 = 不确定/中立 6-8 = 同意 9-10 = 强烈同意

D1. 态度认知 请评价您对以下说法的同意程度。

1. 我认为，手术室工作中产生和使用的塑料制品（如输液 1 2 3 4 5
袋、注射器、包装等）是环境中微塑料污染的重要来源之 6 7 8 9
一。 10

2. 在我看来，日常接触医疗塑料包装（如药品、器械包 1 2 3 4 5
装）对医护人员健康的潜在负面影响微乎其微。 (此为反向 6 7 8 9
计分题) 10

1 2 3 4 5
3. 我认为，在医院和手术室范围内积极减少不必要的塑料
6 7 8 9
消耗，对于保护环境和实现生态可持续发展至关重要。
10

4. 我认为，主动采取措施减少个人微塑料暴露（如自带水 1 2 3 4 5
杯、避免塑料餐盒盛热食）对维护自身健康是值得且有意 6 7 8 9
义的。 10

D2.主观规范 请评价您对以下说法的同意程度。

1 2 3 4 5
1. 我所在科室的多数同事认为，在手术室工作中，使用一
6 7 8 9
次性塑料医疗用品比可重复消毒的器材更为便利和高效。
10

2. 患者或其家属的期望和要求，有时会促使我优先选择使 1 2 3 4 5
用塑料包装的药品或器械（例如，认为这样更安全、更新 6 7 8 9
鲜）。 10

1 2 3 4 5
3. 我身边关系密切的同事（或朋友）的环保行为和习惯，
6 7 8 9
会积极影响我减少使用一次性塑料制品。
10

1 2 3 4 5
4. 我认为，医院或科室管理层会将降低成本和控制感染放
6 7 8 9
在首位，而非优先考虑减少塑料耗材的使用。
10

D3. 感知行为控制 请评价您对以下说法的同意程度。

1. 我觉得自己完全有能力在工作中避免使用大部分一次性塑 1 2 3 4
料制品（例如，坚持自带水杯、餐盒，拒绝不必要的塑料包 5 6 7 8 9
装）。 10

2. 我认为，在手术室繁忙的工作节奏和高压环境下，选择并 1 2 3 4
使用非塑料的替代品（如不锈钢器械盘、玻璃药杯）在操作上 5 6 7 8 9
□ 是便利和可行的。 10

1 2 3 4
3. 我认为，购买和使用环保的、非塑料的日常替代品（如玻
5 6 7 8 9
璃饭盒、不锈钢餐具），不会显著增加我的个人经济负担。
10

4. 当我想减少塑料使用时，我能够方便地获得足够的支持与 1 2 3 4
资源（例如，医院提供直饮水、食堂提供非塑料餐盒选项、容 5 6 7 8 9
易买到环保产品）。 10

D4. 行为意向 指您执行某种行为的意图和意愿的强烈程度。

1. 我计划在未来一个月内，开始或进一步有意识地减少我 1 2 3 4 5
在工作中对一次性塑料耗材的使用量。 6 7 8 9 10

2. 我愿意在条件允许的情况下，主动向科室建议或接受使 1 2 3 4 5
用可重复消毒的容器来替代相应的一次性塑料耗材。 6 7 8 9 10

3. 即使离开工作环境，在家庭和社会生活中，我也会积极 1 2 3 4 5
主动地采取措施，避免接触和购买不必要的塑料制品。 6 7 8 9 10

4. 我愿意花时间和精力去学习如何更好地识别和避免微塑 1 2 3 4 5
料暴露（如学习查看化妆品成分表、了解防护知识）。 6 7 8 9 10

第五部分：健康影响与防护需求 导语： 本部分旨在了解微塑料暴露可能对您健康产生的影响，

并征集您对防护措施的真实需求与建议。您的回答将为改善职业健康环境提供直接依据。

健康影响自评 E1. 症状自评 （多选）

在过去六个月中，您是否出现过以下症状，并怀疑其与工作或生活环境中接触塑料制品有关？（可多选）

□ 皮肤相关：接触塑料制品（如手套、防护服、塑料包装）后出现局部皮肤瘙痒、红疹或过敏。

□ 呼吸道相关：在手术室（特别是电刀使用产生烟雾时）或消毒环境中，感到呼吸道刺激，如咳嗽、胸闷、喉咙不适、鼻塞。

□ 眼部相关：在工作环境中感到眼干、眼涩、发痒或流泪。

□ 消化系统相关：在使用塑料餐盒盛装食物后，出现胃肠不适，如腹痛、腹泻、腹胀。

□ 其他全身症状：如莫名的心悸、血压波动、持续性疲劳或头痛。

□ 未出现上述任何相关症状。

E2. 暴露源重要性排序 如果您的直观感受是工作中的微塑料暴露总体高于生活暴露，请根据您的经验，对以下主要暴露源按贡献度进行排序（将字母填入横线）： 最重要：_____ > 其次：_____ > 第三：_____

NO.______医疗耗材操作（直接处理输液袋、注射器、其他一次性耗材塑料包装开包和使用等）

NO.______防护穿戴（长时间穿戴一次性手术衣、口罩、帽子、手套、鞋套等产生的摩擦与吸入风险）

NO.______饮水餐食（饮料，饮水，打包盒、外卖餐盒、塑料袋盛装食物）

NO.______手术室环境暴露（手术电刀烟雾、消毒剂气溶胶等）

NO.______专科手术配合过程中的暴露（使用骨水泥、脉冲等）

防护行为与需求 E3. 防护行为对比 请比较您在工作场所与居家生活中，执行以下防护行为的频

率。

E3.1 参加本次微塑料问卷前、您是否有意识的主动筛查并避免购买和使用含微塑料的化妆品/
护肤品 （单选）
（如查看成分表，避开聚乙烯、聚丙烯等）

□ 总是如此

□ 有时如此

□ 很少如此

□ 从不注意

E3.2 参加本次微塑料问卷前、工作中（在医院期间）您是否有意识的避免用塑料容器盛装高温食物/液体 （单选）

（如不用塑料盒微波加热、不装热汤）

□ 总是如此
□ 有时如此
□ 很少如此
□ 从不注意

E3.3 参加本次微塑料问卷前、工作外（居家、外出时）您是否有意识的避免用塑料容器盛装高温食物/液体 （单选）

（如不用塑料盒微波加热、不装热汤）

□ 总是如此
□ 有时如此

□ 很少如此
□ 从不注意

E3.4 参加本次微塑料问卷前、工作中（在医院期间）您是否有意识地选择天然材质容器 （单选）

（如优先使用自带的不锈钢/玻璃水杯和餐盒）

□ 总是如此
□ 有时如此
□ 很少如此
□ 从不注意

E3.5 参加本次微塑料问卷前、工作外（居家生活、外出）您是否有意识地选择天然材质容器（单选）

（如优先使用自带的不锈钢/玻璃水杯和餐盒）

□ 总是如此

□ 有时如此
□ 很少如此
□ 从不注意

E4 医院支持需求 您最希望医院或科室提供哪些支持，以帮助您有效减少微塑料暴露？（请勾选您认为最重要的 3-5 项） （多选）

□ 提供可行的非塑料医疗替代品（如在合适场景推广使用玻璃输液瓶、不锈钢器械盒等）
□ 改善基础设施（在手术室休息区等关键区域安装高品质直饮水机，并配发个人专用玻璃杯或
□ 不锈钢杯）
□ 改革食堂供餐模式（食堂全面禁用塑料打包盒，提供可重复清洗、消毒的公共餐盘或租借式餐盒服务）
□ 开展专项知识与技能培训（定期组织关于微塑料健康风险与日常、职业防护措施的专题讲座或培训）

□ 纳入职业健康监测（在年度职业健康体检中，增加与微塑料暴露相关的潜在健康指标筛查和咨询）
□ 提供夜班/值班专项防护支持（为值班人员配备"健康防护包"，内含不锈钢餐具、玻璃饭盒、天然纤维毛巾等）
□ 优化手术室通风与烟雾管理（加强烟雾吸附系统，确保有效去除电刀等产生的有害气溶胶）

□ 其他建议（请简要说明）：________________

第六部分：开放建议 F1 对于在手术室等医疗环境中，如何系统性地、有效地减少护理人员的微

塑料暴露风险，您有何具体的意见或建议？（例如：在管理政策、科室流程、个人防护、设施改造、宣传教育等任何方面的想法都欢迎提出）

____
____
____

第 5 部分：质量控制（注意力检测）请选择“同意” 选项 （单选）

请准确选择“同意”选项，选错将视为无效问卷

□ 完全不同意
□ 不同意
□ 一般
□ 同意
□ 完全同意

问卷全部结束，再次衷心感谢您付出的宝贵时间和提出的真知灼见！您的每一份回答都将为守护医护同仁的职业健康注入一份力量。
