# Supplementary material for: Self‐Reported Plastic‐Related Occupational Scenarios and Perceived Support Priorities Among Operating‐Room Nursing Staff: A Multicentre Cross‐Sectional Survey
Source: J Nurs Manag. 2026 Oct 17;2026:7475740. doi: 10.1155/jonm/7475740 (PMC13675910; doi:10.1155/jonm/7475740)
Supplement: Supplementary file 1 [file JONM-2026-7475740-s001.zip › Supplementary_File_2_English_questionnaire.pdf]

Multicentre Survey of Microplastic Exposure-Related Risk among Operating Room Nursing Staff

English translation of the questionnaire

Translation and confidentiality note

This document is an English translation prepared for manuscript supplementary materials. It follows the structure and content of the Chinese questionnaire used in the study.

Study-site names are anonymised in this English version to protect institutional confidentiality. The site-identification key should not be submitted or shared.

The original survey included a data-checking identifier. Such identifiers were removed before analysis and are not included in shared analysis datasets.

Invitation and informed consent

Dear nursing colleague,

We are the research team conducting a multicentre survey on microplastic exposure-related risk among operating room nursing staff. We sincerely invite you to participate in this study.

Microplastics are small plastic particles widely present in the environment. In operating room environments, nursing staff may be exposed to microplastics through multiple pathways. This study aims to understand the potential exposure-related risk, awareness and protective behaviours of operating room nursing staff. The findings will provide scientific evidence for developing targeted occupational protection strategies and improving occupational health for healthcare workers.

This study was approved by the relevant institutional ethics committee [approval number: 2025-066]. The survey is confidential, and all information will be used only for aggregate analysis. Completing the questionnaire takes approximately 15–20 minutes. Participation is entirely voluntary, but every response is important.

Consent statement

I have read and understood the information above, and my questions have been answered. I voluntarily agree to participate in this study.

□ I confirm that I have read and understood the information above. I voluntarily agree to participate in this questionnaire survey. I understand that I may withdraw my data within 7 days after submission by contacting the research team by email and providing the required withdrawal information.

Part 1. Basic information

All information in this section is important for the study and will be kept confidential. Please answer according to your actual situation.

A1. Current work institution (single choice)

□ Site A

□ Site B

□ Site C

□ Other participating site (please specify): __________

A2. Staff badge number (for data checking only; removed before analysis)

Response: ____

A3. Sex (single choice)

□ Male

□ Female

A4. Age

Age in years: ____

A5. Height and weight (for health indicator analysis)

Item	Value
Height, cm	
Weight, kg	

A6. Years of working experience in the operating room (single choice)

□ <1 year

□ 1–3 years

□ 4–6 years

□ 7–10 years

□ >10 years

A7. Main role(s) in the operating room (multiple choice)

□ Circulating nurse

□ Scrub/instrument nurse

□ Anaesthesia monitoring nurse

□ Other non-surgical role, such as management, billing review, etc. (please specify): __________

A8. Main types of surgery in which you participate (multiple choice; select 1–12 items as applicable)

□ General surgery

□ Orthopaedic surgery

□ Extracorporeal/cardiac surgery

□ Neurosurgery

□ Interventional surgery

□ Gynaecology/urology

□ Otorhinolaryngology

□ Breast and thyroid surgery

□ Thoracic surgery

□ Plastic surgery

□ Ophthalmology

□ Other or non-surgical role (please specify, e.g., billing review/sterile room management/quality control/scheduling): __________

A9. Which of the following best describes your current shift pattern? (single choice)

□ Day-only work, usually administrative daytime work without night duty

□ Rotating shift work, including night shifts, handover shifts, second-line duty, etc.

□ Other (please specify): __________

A9.1. On average, how many night shifts do you have per month? (single choice)

□ Fewer than 5

□ 5 or more

□ Not applicable/no night shift

A10. Highest education level (single choice)

□ Technical secondary school

□ Junior college/diploma

□ Bachelor's degree

□ Postgraduate degree or above

A11. Professional title (single choice)

□ Nurse

□ Senior nurse

□ Supervisor nurse

□ Associate chief nurse or above

Part 2. Awareness and concern regarding microplastics

This section asks about your familiarity with and personal concern about microplastics. There are no right or wrong answers. Please answer according to your actual situation.

For each item, please rate awareness and concern using a 1–10 scale: 1 = not aware / not concerned; 10 = very aware / very concerned.

B1. Basic concepts

B1.1. Before participating in this survey, how aware and concerned were you about "microplastics"?

Microplastics refer to plastic particles, fibres or fragments smaller than 5 mm in the environment, such as particles in exfoliating products or tiny plastic particles that may be present in bottled water.

Awareness: 1 2 3 4 5 6 7 8 9 10

Concern: 1 2 3 4 5 6 7 8 9 10

B1.2. Before participating in this survey, how aware and concerned were you about microplastics in personal care and cosmetic products (PCCPs)?

PCCPs may intentionally contain plastic microbeads or polymer ingredients, such as those in exfoliating cleansers, toothpaste or shower gels. These ingredients may be used for exfoliation, thickening or smoothness and may appear on ingredient labels as polyethylene (PE), polypropylene (PP), polyquaternium and related terms.

Awareness: 1 2 3 4 5 6 7 8 9 10

Concern: 1 2 3 4 5 6 7 8 9 10

B1.3. Before participating in this survey, how aware and concerned were you about microplastic release in healthcare environments?

This refers to the possibility that plastic products release microplastics during medical procedures, such as frequent friction or opening of plastic packaging, syringes or devices, wear of protective clothing or shoe covers, surgical smoke aerosols, and airborne synthetic fibres.

Awareness: 1 2 3 4 5 6 7 8 9 10

Concern: 1 2 3 4 5 6 7 8 9 10

B2. Health risks related to microplastics

B2.1. Before participating in this survey, how aware and concerned were you about inflammatory responses potentially caused by microplastics?

Studies suggest that microplastics entering the human body may abnormally activate immune cells and trigger local or systemic chronic inflammation.

Awareness: 1 2 3 4 5 6 7 8 9 10

Concern: 1 2 3 4 5 6 7 8 9 10

B2.2. Before participating in this survey, how aware and concerned were you about the association between microplastics and cardiovascular disease?

Recent clinical research reported that patients in whom microplastics were detected in carotid plaques had a higher subsequent risk of myocardial infarction, stroke or death than those without detected microplastics.

Awareness: 1 2 3 4 5 6 7 8 9 10

Concern: 1 2 3 4 5 6 7 8 9 10

B3. Protective measures related to microplastics

B3.1. Before participating in this survey, how aware and concerned were you about the potential effect of boiling tap water on removing microplastics?

Boiling tap water followed by filtration has been reported as a simple daily protective method that may reduce nano-/microplastic particles in water.

Awareness: 1 2 3 4 5 6 7 8 9 10

Concern: 1 2 3 4 5 6 7 8 9 10

B3.2. Before participating in this survey, how aware and concerned were you about the value of natural-material containers as alternatives?

Using glass, stainless steel or ceramic containers instead of plastic cups or meal boxes may help reduce the release and ingestion of microplastics from food and drink containers.

Awareness: 1 2 3 4 5 6 7 8 9 10

Concern: 1 2 3 4 5 6 7 8 9 10

Part 3. Assessment of exposure pathways

This section asks about potential microplastic exposure pathways and their frequency in daily life and the operating room occupational environment. Please recall the past month unless otherwise specified.

Daily-life exposure: C1. Drinking-water habits

C1.1. What is the main source of drinking water in your operating room? (single choice)

□ Barrelled water with water dispenser

□ Piped direct drinking water connected to the hospital water-purification system

□ Bottled water only

□ Other (please specify): __________

C1.2. During work, what is the main material of the drinking container you most often use? (single choice)

Please think about the cup or bottle you use most often.

□ Plastic cup/bottle

□ Stainless-steel cup/bottle

□ Glass/ceramic cup

□ Disposable paper cup

□ Varies by situation; no fixed container

C1.3. Outside work, what is the main material of the drinking container you most often use? (single choice)

Please think about the cup or bottle you use most often.

□ Plastic cup/bottle

□ Stainless-steel cup/bottle

□ Glass/ceramic cup

□ Disposable paper cup

□ Varies by situation; no fixed container

C1.4. During the past month at work, how often did you drink plastic bottled water, bottled beverages or takeaway drinks such as milk tea or coffee? (single choice)

□ Multiple times per day

□ Once per day

□ 2–3 times per week

□ Rarely (<1 time per week)

□ Never

C1.5. During the past month outside work, how often did you drink plastic bottled water, bottled beverages or takeaway drinks such as milk tea or coffee? (single choice)

□ Multiple times per day

□ Once per day

□ 2–3 times per week

□ Rarely (<1 time per week)

□ Never

C1.6. During the past month at work, how often did you use disposable paper cups for drinking water? (single choice)

□ Always (almost every time)

□ Often (most of the time)

□ Sometimes (about half of the time)

□ Rarely (occasionally)

□ Never (I only use my own cup)

C1.7. During the past month outside work, how often did you use disposable paper cups for drinking water? (single choice)

□ Always (almost every time)

□ Often (most of the time)

□ Sometimes (about half of the time)

□ Rarely (occasionally)

□ Never (I only use my own cup)

Daily-life exposure: C2. Dietary habits

C2.1. During the past month at work, how often did you use plastic meal boxes, plastic bags or plastic wrap to directly contain hot food or hot dishes? (single choice)

Including microwaving plastic meal boxes or directly containing freshly cooked hot dishes or soup.

□ Very high (>80% of the time)

□ High (50%–80% of the time)

□ Moderate (30%–50% of the time)

□ Low (<30% of the time)

□ Never

C2.2. During the past month outside work, how often did you use plastic meal boxes, plastic bags or plastic wrap to directly contain hot food or hot dishes? (single choice)

Including at home, dining out or ordering takeaway; including microwaving plastic meal boxes or directly containing freshly cooked hot dishes or soup.

□ Very high (>80% of the time)

□ High (50%–80% of the time)

□ Moderate (30%–50% of the time)

□ Low (<30% of the time)

□ Never

Daily-life exposure: C3. Personal care and product-use habits

C3.1. Toothpaste: Do you use toothpaste containing plastic microbeads, often listed as polyethylene (PE) or polypropylene (PP) on the ingredient label? (single choice)

□ Yes, I use it often and know that it contains microbeads

□ I use it often but do not know whether it contains microbeads

□ I intentionally choose toothpaste without microbeads

□ No fixed choice; I have not paid attention to this issue

C3.2. Exfoliating facial cleanser/scrub: How often do you use cleansing products containing plastic exfoliating particles? (single choice)

Product names may include deep-cleaning scrub, exfoliating cleanser or bead cleanser. Ingredient labels may include polyethylene (PE), polypropylene (PP) or polyethylene terephthalate (PET).

□ Daily

□ 3–5 times per week

□ 1–2 times per week

□ Occasionally (a few times per month)

□ Never

Occupational exposure: C4. Operating-room-specific medical exposure assessment

C4.1. In the past month, on average, how often did you handle or process plastic medical items each day? (single choice)

Examples include preparing medications, squeezing infusion bags, connecting/disconnecting syringes or extension tubes, three-way stopcocks, etc.

□ Very little contact

□ Moderate (<10 times/day)

□ High (<20 times/day)

□ Frequent (20–30 times/day)

□ Very frequent (>30 times/day)

C4.2. In the past month, on average, what was your cumulative duration of wearing disposable surgical gowns during each day shift? (single choice)

□ Almost never wear them

□ <2 hours

□ 2–4 hours (including 2 hours)

□ 4–6 hours (including 4 hours)

□ >6 hours

C4.3. How often do you wear disposable surgical caps at work? (single choice)

□ Always

□ Often

□ Sometimes

□ Occasionally

□ Almost never

C4.4. In the past month, on average, how many plastic outer packages of disposable sterile items did you open during each day shift? (single choice)

Examples include dressing packs, instrument packs and various consumables.

□ Few (1–30)

□ Many (31–60)

□ Very many (61–90)

□ Extremely many (>90)

□ Unfixed; highly variable/difficult to estimate

C4.5. In the past month, on average, for how many operations did you perform surgical draping during each day shift? (single choice)

□ Few (1–2 operations)

□ Moderate (3–5 operations)

□ Many (5–9 operations)

□ Extremely many (10 or more operations)

□ Not involved

C4.6. In the past month, on average, how many pairs of disposable film gloves or PVC gloves did you use during each day shift? (single choice)

□ 5 pairs or more

□ 3–4 pairs

□ 1–2 pairs

□ 0 pairs (use other protection or operate directly)

C4.7. In the past month, among the operations in which you participated, how often did energy devices cause visible or odorous smoke due to burning of plastic instruments or dressings? (single choice)

This mainly refers to smoke generated during the use of electrocautery, ultrasonic scalpels and other energy devices. High temperature may cause pyrolysis or vaporisation of human tissue and contacting plastic instruments, dressings or packaging, potentially including vaporised plastic components.

□ Often (in ≥50% of operations)

□ Sometimes (in about 10%–50% of operations)

□ Occasionally (<10% of operations)

□ Almost never

□ Not involved in such operations

C4.8. During open surgery, when electrocautery, ultrasonic scalpel or similar devices produce obvious smoke, how often can your team immediately use suction or an efficient smoke evacuator? (single choice)

□ Always immediately available

□ Often immediately available

□ Available in about half of cases

□ Rarely immediately available

□ Almost never used/equipment is available but response is not timely

C4.9. During laparoscopic surgery, when electrocautery, ultrasonic scalpel or similar devices produce obvious smoke, how does your team usually manage it? (single choice)

□ Routine active protection: use negative-pressure suction or an automatic smoke evacuator connected through the trocar valve

□ Frequent active protection: often use negative-pressure venting, but sometimes it may be overlooked

□ Mixed use depending on situation: sometimes suction is used and sometimes smoke is directly released into room air depending on smoke volume and equipment

□ Routine passive release: usually directly open the valve and release smoke into the operating-room air

□ Not involved/no fixed mode: do not perform this type of surgery or the method depends entirely on the surgeon's habit

C4.10. In the past month, during surgical cooperation, how often were you involved in operations using bone cement, pulse lavage or related procedures? (single choice)

□ At least once per day

□ 2–4 times per week

□ Once per week

□ Occasionally (a few times per month)

□ Almost not involved

Hospital support and logistical support in the operating room

C5.1. What is the main meal-provision model for on-duty staff in your operating room/hospital? (single choice)

□ Unified meals from the hospital canteen, packed in hospital-provided meal boxes and delivered to the operating room

□ Customised takeaway service through a contracted catering company

□ No unified arrangement; entirely self-managed (order takeaway or bring meals yourself)

□ Mixed model (briefly describe): ___________

C5.2. What do you think is the main filling material of quilts and pillows provided in the duty room? (single choice)

□ Synthetic fibre cotton, such as hollow fibre or PP cotton

□ Natural materials, such as cotton or down

□ Not sure

□ Not provided

C5.3. What type of caps are routinely provided for healthcare staff in your operating room? (single choice)

□ Disposable non-woven caps (most common; various colours; relatively stiff texture)

□ Disposable medical paper caps (paper-like texture, breathable, easily creased)

□ Reusable pure cotton cloth caps, uniformly cleaned and disinfected by the hospital

□ Reusable synthetic-fibre cloth caps, such as polyester

□ Mixed provision or no fixed type

Part 4. Attitudes and behavioural intention

This section asks about your views, feelings and plans regarding reducing microplastic exposure. Please indicate your agreement with each statement. There are no right or wrong answers.

Use a 1–10 scale: 1 = strongly disagree; 2–3 = disagree; 4–5 = uncertain/neutral; 6–8 = agree; 9–10 = strongly agree.

D1. Attitude

D1.1. I believe that plastic products generated and used in operating-room work, such as infusion bags, syringes and packaging, are one of the important sources of microplastic pollution in the environment.

Agreement: 1 2 3 4 5 6 7 8 9 10

D1.2. In my view, daily contact with medical plastic packaging, such as medicine or instrument packaging, has negligible potential negative health effects for healthcare workers. (Reverse-scored item)
Agreement: 1 2 3 4 5 6 7 8 9 10

D1.3. I believe that actively reducing unnecessary plastic consumption within hospitals and operating rooms is essential for environmental protection and ecological sustainability.
Agreement: 1 2 3 4 5 6 7 8 9 10

D1.4. I believe that taking active measures to reduce my personal microplastic exposure, such as bringing my own cup or avoiding hot food in plastic containers, is worthwhile and meaningful for maintaining my own health.
Agreement: 1 2 3 4 5 6 7 8 9 10

D2. Subjective norms

D2.1. Most colleagues in my department believe that, in operating-room work, disposable plastic medical supplies are more convenient and efficient than reusable sterilised equipment.
Agreement: 1 2 3 4 5 6 7 8 9 10

D2.2. The expectations and requests of patients or family members sometimes lead me to prioritise medicines or instruments with plastic packaging, for example because they are perceived as safer or newer.
Agreement: 1 2 3 4 5 6 7 8 9 10

D2.3. The environmentally friendly behaviours and habits of close colleagues or friends around me positively influence me to reduce the use of disposable plastic products.
Agreement: 1 2 3 4 5 6 7 8 9 10

D2.4. I believe that hospital or department management prioritises cost reduction and infection control rather than reducing the use of plastic consumables.
Agreement: 1 2 3 4 5 6 7 8 9 10

D3. Perceived behavioural control

D3.1. I feel fully capable of avoiding the use of most disposable plastic products at work, for example by bringing my own cup and meal box and refusing unnecessary plastic packaging.
Agreement: 1 2 3 4 5 6 7 8 9 10

D3.2. I think that, in the busy and high-pressure operating-room environment, choosing and using non-plastic alternatives, such as stainless-steel instrument trays or glass medicine cups, is convenient and feasible in practice.
Agreement: 1 2 3 4 5 6 7 8 9 10

D3.3. I think that purchasing and using environmentally friendly non-plastic daily alternatives, such as glass meal boxes and stainless-steel tableware, would not substantially increase my personal financial burden.
Agreement: 1 2 3 4 5 6 7 8 9 10

D3.4. When I want to reduce plastic use, I can conveniently obtain sufficient support and resources, such as direct drinking water provided by the hospital, non-plastic meal-box options from the canteen, or easy access to environmentally friendly products.
Agreement: 1 2 3 4 5 6 7 8 9 10

D4. Behavioural intention

Behavioural intention refers to the strength of your intention and willingness to perform a behaviour.

D4.1. I plan to begin or further consciously reduce my use of disposable plastic consumables at work within the next month.
Agreement: 1 2 3 4 5 6 7 8 9 10

D4.2. When conditions allow, I am willing to actively suggest to my department or accept the use of reusable sterilised containers to replace corresponding disposable plastic consumables.

Agreement: 1 2 3 4 5 6 7 8 9 10

D4.3. Even outside the work environment, in family and social life, I will actively take measures to avoid contact with and purchase of unnecessary plastic products.

Agreement: 1 2 3 4 5 6 7 8 9 10

D4.4. I am willing to spend time and effort learning how to better identify and avoid microplastic exposure, such as learning to read cosmetic ingredient labels and understand protective knowledge.

Agreement: 1 2 3 4 5 6 7 8 9 10

Part 5. Health effects and protective needs

This section asks about possible health effects related to microplastic exposure and your needs and suggestions for protective measures. Your answers will provide direct evidence for improving occupational health environments.

Self-rated health effects

E1. Symptom self-assessment (multiple choice)

In the past 6 months, have you experienced any of the following symptoms and suspected that they were related to contact with plastic products in the work or living environment?

- □ Skin-related symptoms: local skin itching, rash or allergy after contact with plastic products such as gloves, protective clothing or plastic packaging.
- □ Respiratory symptoms: respiratory irritation in the operating room, especially when electrocautery smoke is generated, or in disinfection environments, such as cough, chest tightness, throat discomfort or nasal congestion.
- □ Eye-related symptoms: dry, gritty, itchy or watery eyes in the work environment.
- □ Digestive-system symptoms: gastrointestinal discomfort after food is placed in plastic meal boxes, such as abdominal pain, diarrhoea or bloating.
- □ Other systemic symptoms, such as unexplained palpitations, blood-pressure fluctuation, persistent fatigue or headache.
- □ None of the above related symptoms.

E2. Ranking of exposure-source importance

If your intuitive feeling is that microplastic exposure at work is generally higher than exposure in daily life, please rank the following main exposure sources according to their contribution based on your experience. Fill in the letters on the lines: Most important: ____ > Second: ____ > Third: ____.

- □ A. Medical consumable operations, such as handling infusion bags, syringes and other disposable consumable plastic packaging and use.
- □ B. Protective clothing and equipment, such as long-term wearing of disposable surgical gowns, masks, caps, gloves and shoe covers, with friction and inhalation risk.
- □ C. Drinking water and meals, such as beverages, drinking water, takeaway meal boxes and plastic bags used to contain food.
- □ D. Operating-room environmental exposure, such as electrocautery smoke and disinfectant aerosols.
- □ E. Exposure during specialty surgical cooperation, such as use of bone cement and pulse lavage.

Protective behaviours and needs

E3. Protective behaviour comparison

Please compare the frequency with which you perform the following protective behaviours at work and in daily life. Response options: Always; Sometimes; Rarely; Never pay attention.

E3.1. Before participating in this survey, did you consciously screen for and avoid buying or using cosmetics/skincare products containing microplastics?

For example, checking ingredient labels and avoiding polyethylene or polypropylene.

□ Always

□ Sometimes

□ Rarely

□ Never pay attention

E3.2. Before participating in this survey, during work, did you consciously avoid using plastic containers for high-temperature food or liquids?

For example, not microwaving plastic boxes or using them for hot soup.

□ Always

□ Sometimes

□ Rarely

□ Never pay attention

E3.3. Before participating in this survey, outside work, did you consciously avoid using plastic containers for high-temperature food or liquids?

For example, not microwaving plastic boxes or using them for hot soup.

□ Always

□ Sometimes

□ Rarely

□ Never pay attention

E3.4. Before participating in this survey, during work, did you consciously choose natural-material containers?

For example, prioritising your own stainless-steel or glass cup and meal box.

□ Always

□ Sometimes

□ Rarely

□ Never pay attention

E3.5. Before participating in this survey, outside work, did you consciously choose natural-material containers?

For example, prioritising your own stainless-steel or glass cup and meal box.

□ Always

□ Sometimes

□ Rarely

□ Never pay attention

E4. Institutional support needs

Which supports would you most like the hospital or department to provide to help you effectively reduce microplastic exposure? Please select the 3–5 items you consider most important.

□ Provide feasible non-plastic medical alternatives, such as promoting glass infusion bottles or stainless-steel instrument boxes in suitable scenarios.

□ Improve infrastructure, such as installing high-quality direct drinking water machines in operating-room rest areas and providing personal glass or stainless-steel cups.

□ Reform the canteen meal-provision model, such as fully eliminating plastic takeaway meal boxes and providing reusable washable and disinfectable public plates or rental meal-box services.

□ Provide specialised knowledge and skills training, such as regular lectures or training on microplastic health risks and daily/occupational protection measures.

□ Include microplastic-related concerns in occupational health monitoring, such as adding screening and consultation for potential health indicators related to microplastic exposure during annual occupational health examinations.

□ Provide special protective support for night-duty/on-duty staff, such as a "health protection package" containing stainless-steel tableware, glass meal boxes and natural-fibre towels.

□ Optimise operating-room ventilation and smoke management, such as strengthening smoke-adsorption systems to effectively remove harmful aerosols generated by electrocautery and related devices.

□ Other suggestions (briefly describe): __________

Part 6. Open-ended suggestions

F1. What specific opinions or suggestions do you have for systematically and effectively reducing microplastic exposure-related risk among nursing staff in operating rooms or other healthcare environments?

Examples may include management policy, department workflow, personal protection, facility improvement, education or any other relevant ideas.

Open-ended response: ____

____

____

____

Quality-control item (attention check)

Please select "Agree". (single choice)

Please accurately select "Agree". Selecting a different option will be regarded as an invalid questionnaire response.

□ Strongly disagree

□ Disagree

□ Neutral

□ Agree

□ Strongly agree

The questionnaire is now complete. Thank you again for your valuable time and thoughtful responses. Every response contributes to protecting the occupational health of healthcare colleagues.
